# Ketamine, Etomidate, and Mortality in Emergency Department Intubations

**DOI:** 10.1001/jamanetworkopen.2025.48060

**Published:** 2025-12-15

**Authors:** Ian Ward A. Maia, Sérgio R. R. Decker, Lucas Oliveira J. e Silva, Rafael von Hellmann, Julio C. G. Alencar, Ludhmila Abrahao Hajjar, Julia M. Dorn de Carvalho, Daniel F. Pedrollo, Caio G. Nogueira, Natalia Mansur P. Figueiredo, Carlos Henrique Miranda, Danilo Martins, Thiago D. Baumgratz, Bruno Bergesch, Osmar Colleoni, Juliana Zanettini, Ana Paula Freitas, Renato Tambelli, Maria Cristina Costa, Wilsterman Correia, Rafael G. de Maria, Ubirajara A. V. Filho, Andre P. Weber, Vinicius da Silva Castro, Carlos Fernando D. Dornelles, Barbara S. Tabach, Nicole P. Moreira, Patricia L. Gaspar, Hélio P. Guimarães, Gabriela Stanzani, Thiago F. Gava, Aidan Mullan, Caitlin S. Brown, Fernanda Bellolio, Molly M. Jeffery, Otavio T. Ranzani, Bruno A. M. P. Besen

**Affiliations:** 1 Department of Emergency Medicine, Faculdade de Medicina da Universidade de Sao Paulo, Sao Paulo, Brazil; 2 Department of Emergency Medicine, Mayo Clinic, Rochester, Minnesota; 3 Outcomes Research Lab, MOVE, Hospital Moinhos de Vento, Porto Alegre, Brazil; 4 Richard A. and Susan F. Smith Center for Outcomes Research, Beth Israel Deaconess Medical Center and Harvard Medical School, Boston, Massachusetts; 5 Department of Emergency Medicine, Hospital de Clinicas de Porto Alegre, Porto Alegre, Rio Grande do Sul, Brazil; 6 Department of Emergency Medicine, Monash Health, Melbourne, Victoria, Australia; 7 Universidade de Sao Paulo, Faculdade de Medicina de Bauru, Bauru, Brazil; 8 Department of Emergency Medicine, Hospital Metropolitano Odilon Behrens, Belo Horizonte, Minas Gerais, Brazil; 9 Department of Emergency Medicine, Ribeirao Preto School of Medicine, University of Sao Paulo, Ribeirao Preto, São Paulo, Brazil; 10 Department of Emergency Medicine, Universidade Estadual Paulista, Botucatu, Sao Paulo, Brazil; 11 Department of Emergency Medicine, Hospital Regional de Sao Jose Doutor Homero de Miranda Gomes, Florianópolis, Santa Catarina, Brazil; 12 Department of Emergency Medicine, Hospital Sao Lucas da Pontificia Universidade Católica do Rio Grande do Sul, Porto Alegre, Rio Grande do Sul, Brazil; 13 Department of Emergency Medicine, Hospital de Pronto Socorro, Porto Alegre, Rio Grande do Sul, Brazil; 14 Department of Emergency Medicine, Hospital das Clínicas da Faculdade de Medicina de Marilia, Marilia, Sao Paulo, Brazil; 15 Department of Emergency Medicine, Hospital Augusto de Oliveira Camargo, Indaiatuba, Sao Paulo, Brazil; 16 Department of Emergency Medicine, Hospital Regional Alto Vale, Rio do Sul, Santa Catarina, Brazil; 17 Department of Emergency Medicine, Hospital Santo Antonio, Sinop, Mato Grosso, Brazil; 18 Department of Emergency Medicine, Hospital Nossa Senhora da Conceição, Porto Alegre, Rio Grande do Sul, Brazil; 19 Department of Emergency Medicine, Hospital Bruno Born, Lajeado, Rio Grande do Sul, Brazil; 20 Unidade de Pronto Atendimento de Lajeado, Lajeado, Rio Grande do Sul, Brazil; 21 Department of Emergency Medicine, Hospital Santa Cruz, Santa Cruz, Rio Grande do Sul, Brazil; 22 Department of Emergency Medicine, Hospital Geral de Fortaleza, Fortaleza, Ceara, Brazil; 23 Department of Emergency Medicine, Hospital Dr. Carlos Alberto Studart Gomes, Fortaleza, Ceara, Brazil; 24 DataHealth Lab, Institut de Recerca Sant Pau (IR SANT PAU), Barcelona, Spain; 25 Pulmonary Division, Heart Institute, Hospital das Clinicas HCFMUSP, Faculdade de Medicina, Universidade de Sao Paulo, Sao Paulo, Brazil; 26 Intensive Care Department, IDOR Education and Research Institute, São Paulo, São Paulo, Brazil

## Abstract

**Importance:**

The choice of induction agent during rapid sequence intubation (RSI) of critically ill adults may affect clinical outcomes. Although ketamine and etomidate are frequently used for RSI, their comparative effectiveness remains controversial.

**Objective:**

To compare the safety of etomidate vs ketamine for emergency RSI in critically ill adults.

**Design, Setting, and Participants:**

This cohort study used a target trial emulation with observational data collected between March 1, 2022, and April 30, 2024, from 18 emergency departments across Brazil, with data from the Brazilian Airway Registry Cooperation. Adults who underwent RSI and received either etomidate or ketamine as a sole hypnotic agent were included; those with preintubation cardiac arrest or immediate postintubation transfer were excluded.

**Exposure:**

Administration of etomidate or ketamine as the induction agent for RSI.

**Main Outcomes and Measures:**

The primary outcome was 28-day in-hospital mortality. Secondary outcomes included 7-day in-hospital mortality, first-attempt intubation success, and major adverse events (new hemodynamic instability, severe hypoxemia, and cardiac arrest) within 30 minutes after intubation. Inverse probability of treatment weighting was used to adjust for confounding. Risk ratios (RRs) and risk differences (RDs) with 95% CIs were used to compare outcomes between groups.

**Results:**

Among 1810 patients (median age, 64 years [IQR, 50-74 years]; 1048 men [57.9%]), 514 received ketamine and 1296 received etomidate. The median shock index was higher in the ketamine group than the etomidate group (0.81 [IQR, 0.65-1.01] vs 0.76 [IQR, 0.59-0.99]), and preintubation vasopressor use was more common in the ketamine group than the etomidate group (191 of 514 [37.2%] vs 391 of 1296 [30.2%]). Weighted 28-day mortality was higher with etomidate than ketamine (60.5% [95% CI, 57.2%-63.8%] vs 54.4% [95% CI, 45.0%-63.9%]; RR, 1.14 [95% CI, 1.03-1.27]; RD, 7.6% [95% CI, 2.0%-13.3%]). Seven-day mortality was also higher with etomidate than ketamine (35.2% [95% CI, 32.0%-38.3%] vs 30.1% [95% CI, 23.5%-36.7%]; RR, 1.19 [95% CI, 1.04-1.35]). New hemodynamic instability within 30 minutes after intubation was more frequent with ketamine (24.2% [95% CI, 20.4%-28.0%] vs 18.9% [95% CI, 16.7%-21.0%]; RR, 0.78 [0.64-0.95]). There were no statistically significant differences in the other secondary outcomes.

**Conclusions and Relevance:**

In this cohort study of critically ill adults undergoing RSI, etomidate use was associated with higher in-hospital mortality at 7 and 28 days compared with ketamine. These findings highlight the need for definitive randomized clinical trials to compare both agents.

## Key Points

**Question:**

Among critically ill adults undergoing rapid sequence intubation, is etomidate use associated with higher in-hospital mortality than ketamine?

**Findings:**

In this cohort study using target trial emulation with inverse probability of treatment weighting and data from 18 Brazilian emergency departments, etomidate was associated with significantly higher risks of in-hospital mortality at 28 days (60.5% vs 54.4%) and 7 days (35.2% vs 30.1%) compared with ketamine.

**Meaning:**

These findings suggest that etomidate may increase the risk of death and support reevaluating its role in emergency airway management for critically ill adults.

## Teaser

This cohort study compares in-hospital mortality with etomidate vs ketamine for emergency rapid sequence intubation in critically ill adults.

## Introduction

Rapid sequence intubation (RSI) is the standard approach to emergency airway management in critically ill patients.^1^ The choice of induction agent during RSI may influence hemodynamic stability and clinical outcomes, including peri-intubation cardiovascular collapse and mortality.^2,3^ Among available induction agents, etomidate and ketamine are frequently chosen due to their favorable pharmacologic profiles.^4,5^

Etomidate, traditionally favored because of minimal cardiovascular depression and relative hemodynamic stability, is currently the most commonly used induction agent for RSI in emergency departments (EDs) in Brazil^1^ and other countries.^3,6^ However, even a single dose of etomidate transiently inhibits adrenal corticosteroid synthesis through blockade of 11β-hydroxylase, raising concerns regarding increased vasopressor requirements.^2,4^ In contrast, ketamine, an *N*-methyl-d-aspartate receptor antagonist with sympathomimetic properties, is frequently chosen due to its sympathomimetic properties, although peri-intubation hypotension may occur, especially among patients experiencing prolonged shock or catecholamine depletion.^5^ Randomized clinical trials comparing the 2 agents have reported inconsistent findings, often limited by insufficient statistical power.^7,8^ However, emerging observational studies suggest that etomidate use may be associated with higher mortality.^9^ Despite uncertainty, current airway management guidelines suggest that either agent may be used for emergency intubations.^9,10^

To address this growing uncertainty on the potential harms associated with etomidate, we conducted a target trial emulation using observational data from a multicenter Brazilian cohort study to compare etomidate vs ketamine for ED intubations and evaluated their association with 28-day in-hospital mortality in critically ill patients.

## Methods

### Study Design and Data Source

We conducted a target trial emulation^11^ using the target trial emulation framework and data from the Brazilian Airway Registry Cooperation (BARCO), a prospective, multicenter cohort of critically ill adults undergoing emergency intubation across 18 Brazilian EDs between March 1, 2022, and April 30, 2024, with patients followed up until hospital discharge or up to 28 days. Institutional review boards at all participating centers (eAppendix in Supplement 1) approved the protocol, granting a waiver of informed consent due to the observational nature and minimal risk of the study, as well as the use of deidentified data.^1^ We report this study following the Strengthening the Reporting of Observational Studies in Epidemiology (STROBE) reporting guideline for cohort studies and recent recommendations for reporting causal inference studies from observational data.^12,13^

### Target Trial Emulation Specification

We emulated a hypothetical randomized clinical trial among critically ill adults undergoing RSI in the ED to evaluate whether etomidate vs ketamine, as the induction agent for RSI, is associated with an increased risk of in-hospital death at up to 28 days of follow-up. The design components of the target trial and its emulation, including eligibility criteria, treatment strategies, treatment assignment, follow-up, outcome definitions, and causal contrast, are detailed in eTable 1 in Supplement 1.

### Participants

Eligible individuals in the emulated trial included adults aged 18 years or older who underwent intubation in the ED, received either ketamine or etomidate as the sole hypnotic agent, and received a neuromuscular blocker. We excluded patients who experienced cardiac arrest before intubation or were transferred to another hospital after intubation.

### Treatment Strategies, Assignment, and Start of Follow-Up

The intervention strategy was the administration of etomidate compared with ketamine as the primary sedative agent. Treatment assignment occurred at a single well-defined time point and, by design, eligibility, treatment assignment, and start of follow-up occurred at the same time. We emulated randomization with inverse probability of treatment weighting (IPTW) accounting for several baseline covariates and RSI covariates. Follow-up began at the time of intubation and continued until hospital discharge, death, or 28 days after intubation, whichever occurred first.

### Outcomes

The primary safety outcome was the risk of in-hospital death measured at 28 days. Secondary safety outcomes included in-hospital mortality measured at 7 days, first-attempt intubation success, and major adverse events measured within 30 minutes after intubation: severe hypoxemia (peripheral oxygen saturation [Spo_2_] <80%), new hemodynamic instability (defined as systolic arterial pressure <65 mm Hg recorded at least once, new requirement for or increased dose of vasopressors, or administration of a fluid bolus >15 mL/kg to maintain target blood pressure), and peri-intubation cardiac arrest.

### Covariates

The BARCO Registry used standardized, prospective data collection that included detailed documentation of clinical, procedural, and operator-level information.^1^ For this analysis, we extracted demographic characteristics (age, sex, and body mass index [BMI]), comorbidities (age-weighted Charlson Comorbidity Index), organ dysfunction severity (Sequential Organ Failure Assessment [SOFA] score), preintubation physiology (Spo_2_, shock index, vasopressor use, and intravenous fluid administration), procedural data (indication for intubation, timing from decision to procedure, neuromuscular blockade, airway difficulty, and the device used for the first attempt) and operator-level characteristics (specialty, postgraduate year, and years of experience).

### Statistical Analysis

Baseline characteristics for patients receiving ketamine vs those receiving etomidate were described using median (IQR) values for continuous variables and counts (percentages) for categorical variables. Absolute standardized mean differences (SMDs) were used to assess covariate balance before and after weighting. We considered an SMD less than 0.1 as evidence of adequate covariate balance.^14^ All analyses were performed after completion of data collection in April 2024.

To estimate the average treatment effect, we used stabilized^14^ IPTW accounting for (1) patient characteristics (age, sex, BMI, age-weighted Charlson Comorbidity Index, preintubation shock index, SOFA score, and preintubation Spo_2_); (2) intubation-related factors (indication, specific indication for trauma or sepsis, subjective impression of difficult intubation, Cormack-Lehane score, time from indication to intubation, administration of fluids or vasopressors before intubation, induction analgesia, induction paralytics, and first intubation viewing device); and (3) operator characteristics (operator age, years of experience, and specialty). We truncated weights at the 1st and 99th percentiles to reduce the influence of extreme values.^14^ Using these weights, we computed marginal risks, risk ratios (RRs), and risk differences (RDs) for all outcomes.

The primary outcome, 28-day in-hospital mortality, was analyzed using pooled logistic regression after weighting to derive effect estimates. We generated weighted Kaplan-Meier curves to visualize cumulative mortality over time. Secondary outcomes, including 7-day in-hospital mortality, first-attempt intubation success, severe hypoxemia (Spo_2_ <80%), new hemodynamic instability (vasopressor or fluid initiation after intubation), and peri-intubation cardiac arrest, were analyzed using generalized linear models after weighting. Log-binomial models were preferred for estimating RRs, while identity-linked binomial models were used for calculating RDs.^15^ Confidence intervals for RRs were computed using robust (Eicker-Huber-White) standard errors; and for RDs, bootstrap resampling with 5000 replications. Missing data for patient BMI and shock index were imputed using random forest multiple imputation. Patient age, sex, BMI, Charlson Comorbidity Index, SOFA score, shock index, preintubation Spo_2_, and death within 28 days were all included as variables during imputation.^16^ Data were imputed across 10 datasets with propensity scoring and analysis performed using each dataset separately (eFigure 3 and eTable 3 in Supplement 1). Results derived from each imputed dataset were aggregated following the Rubin rules.^17^

We conducted 3 predefined sensitivity analyses to assess the robustness of our primary findings. (1) To account for hospital-level clustering, we reestimated the IPTW including a random intercept for hospital. (2) To address potential violations of the positivity assumption, we excluded centers in which fewer than 10 patients received ketamine, ensuring a minimum level of within-center treatment variability. (3) To evaluate the potential for unmeasured confounding, we calculated E-values for the observed RRs for 28-day mortality. The E-value quantifies the minimum strength of association that an unmeasured confounder would need to have with both the treatment and the outcome to fully explain away the observed association.^18^ Each sensitivity analysis was conducted across the multiple imputed datasets using the same IPTW approach as the primary analysis.

All analyses were performed using R, version 4.3.2 (R Project for Statistical Computing). Results are presented with point estimates, 95% CIs, and *P* values interpreted to the nominal 2-sided .05 value, with no adjustments for multiple comparisons, as this is not a confirmatory clinical trial.

## Results

### Study Population and Baseline Characteristics

Among 2846 patients enrolled in the BARCO registry, 1810 (median age, 64 years [IQR, 50-74 years]; 1048 men [57.9%] and 762 women [42.1%]) were included after exclusions (Table 1; eFigure 1 in Supplement 1): 1296 (71.6%) received etomidate and 514 (28.4%) received ketamine. Detailed demographic and clinical characteristics for the full cohort are shown in Table 1 with SMDs presented before and after weighting. The median dose of etomidate was 0.2 mg/kg (IQR, 0.2-0.3 mg/kg) and the median dose of ketamine was 1.5 mg/kg (IQR, 1.2-2.0 mg/kg). The median shock index was higher in the ketamine group compared with the etomidate group (0.81 [IQR, 0.65-1.01] vs 0.76 [IQR, 0.59-0.99]). Preintubation vasopressor use was less frequent among patients receiving etomidate (391 of 1296 [30.2%]) than among those receiving ketamine (191 of 514 [37.2%]). The median Charlson Comorbidity Index was 3 (IQR, 1-5) in both groups and the median SOFA score was 4 (IQR, 3-6) in the etomidate group and 4 (IQR, 2-7) in the ketamine group. Use of ketamine varied substantially across the 18 participating hospitals, with a median of 15.5% (IQR, 9.9%-40.8%; range, 0.0%-70.6%) of intubations (eTable 2 in Supplement 1). Imbalances observed in the unweighted data were substantially attenuated after application of stabilized IPTW (Table 1). Overall, 302 patients were intubated in urgent care centers or emergency departments without available on-site intensive care unit beds and were subsequently transferred to other hospitals (eFigure 1 in Supplement 1).

**Table 1.** Patient Demographic and Clinical Characteristics and Outcomes

Characteristic	All patients (N = 1810)	Etomidate (n = 1296)	Ketamine (n = 514)	ASMD^a^	
				Unweighted	Weighted
Patient characteristics					
Age, median (IQR), y	64 (50-74)	63 (50-73)	64 (50-74)	0.046	0.018
Sex, No. (%)					
Male	1048 (57.9)	773 (59.6)	275 (53.5)	0.124	0.029
Female	762 (42.1)	523 (40.4)	239 (46.5)		
BMI, median (IQR)	25.7 (23.4-27.8)	25.7 (23.4-27.9)	25.0 (22.9-27.8)	0.043	0.012
Shock index, median (IQR)	0.78 (0.60-0.99)	0.76 (0.59-0.99)	0.81 (0.65-1.01)	0.131	0.064
Charlson Comorbidity Index, median (IQR)	3 (1-5)	3 (1-5)	3 (1-5)	0.093	0.009
SOFA score, median (IQR)	4 (3-6)	4 (3-6)	4 (2-7)	0.049	0.003
Hemodynamic resuscitation before intubation, No. (%)					
Fluids	399 (22.0)	276 (21.3)	123 (23.9)	0.063	0.056
Vasopressors	582 (32.2)	391 (30.2)	191 (37.2)	0.148	0.015
Blood transfusion	50 (2.8)	39 (3.0)	11 (2.1)	0.055	0.033
Intubation characteristics					
Indication for intubation, No. (%)					
Airway protection	853 (47.1)	668 (51.5)	185 (36.0)	0.317	0.080
Acute respiratory failure	630 (34.8)	400 (30.9)	230 (44.7)	0.289	0.059
Anticipation of clinical course	290 (16.0)	198 (15.3)	92 (17.9)	0.071	0.024
Transport risk	9 (0.5)	9 (0.7)	0	0.118	0.091
Not recorded	28 (1.5)	21 (1.6)	7 (1.4)	0.021	0.069
Primary diagnosis of trauma, No. (%)	142 (7.8)	114 (8.8)	28 (5.4)	0.130	0.018
Primary diagnosis of sepsis, No. (%)	132 (7.3)	84 (6.5)	48 (9.3)	0.106	0.011
Subjective impression of difficult intubation, No. (%)	483 (26.7)	366 (28.2)	117 (22.8)	0.126	0.032
Time between indication and intubation, No. (%)					
0-15 min	825 (45.6)	606 (46.8)	219 (42.6)	0.084	0.019
16-60 min	819 (45.2)	585 (45.1)	234 (45.5)	0.008	0.009
>60 min	163 (9.0)	102 (7.9)	61 (11.9)	0.134	0.026
Unknown timing	3 (0.2)	3 (0.2)	0	0.068	0.061
Spo_2_ measured before intubation, median (IQR), %	99 (96-100)	99 (96-100)	99 (95-100)	0.021	0.009
First attempt device, No. (%)					
DL–curved	1430 (79.0)	1070 (82.6)	360 (70.0)	0.298	0.005
VL–standard	315 (17.4)	179 (13.8)	136 (26.5)	0.319	0.012
VL–hyperangulated	48 (2.7)	34 (2.6)	14 (2.7)	0.006	0.012
Other device	16 (0.9)	12 (0.9)	4 (0.8)	0.016	0.001
None	1 (0.1)	1 (0.1)	0	0.039	0.030
Auxiliary intubation device, No. (%)					
Stylet	990 (54.7)	792 (61.1)	198 (38.5)	0.464	0.041
Bougie	574 (31.7)	319 (24.6)	255 (49.6)	0.536	0.047
Other	3 (0.2)	2 (0.2)	1 (0.2)	0.010	0.019
None	241 (13.3)	182 (14.0)	59 (11.5)	0.077	0.047
Unknown or not recorded	2 (0.1)	1 (0.1)	1 (0.2)	0.032	0.041
Cormack-Lehane classification, No. (%)					
1	845 (46.7)	592 (45.7)	253 (49.2)	0.071	0.012
2a	541 (29.9)	396 (30.6)	145 (28.2)	0.052	0.006
2b	254 (14.0)	186 (14.4)	68 (13.2)	0.033	0.035
3	96 (5.3)	70 (5.4)	26 (5.1)	0.015	0.003
4	9 (0.5)	5 (0.4)	4 (0.8)	0.052	0.007
Not available	65 (3.6)	47 (3.6)	18 (3.5)	0.067	0.014
Induction analgesia, No. (%)					
Fentanyl	326 (18.0)	294 (22.7)	32 (6.2)	0.481	0.080
Lidocaine	36 (2.0)	23 (1.8)	13 (2.5)	0.052	0.042
Other	53 (2.9)	31 (2.4)	22 (4.3)	0.105	0.000
None	1395 (77.1)	948 (73.1)	447 (87.0)	0.351	0.057
Induction neuromuscular blocker, No. (%)					
Succinylcholine	1070 (59.1)	812 (62.7)	258 (50.2)	0.253	0.005
Rocuronium	734 (40.6)	478 (36.9)	256 (49.8)	0.263	0.002
Other	4 (0.2)	4 (0.3)	0	0.079	0.055
Unknown	2 (0.1)	2 (0.2)	0	0.056	0.039
First intubation operator characteristics					
Operator specialty, No. (%)					
Emergency medicine	720 (39.8)	524 (40.4)	196 (38.1)	0.047	0.004
Internal medicine	707 (39.1)	500 (38.6)	207 (40.3)	0.035	0.029
Surgery	106 (5.9)	79 (6.1)	27 (5.3)	0.036	0.040
Medical student	59 (3.3)	37 (2.9)	22 (4.3)	0.077	0.036
ICU	26 (1.4)	17 (1.3)	9 (1.8)	0.036	0.015
Anesthesiology	2 (0.1)	1 (0.1)	1 (0.2)	0.032	0.002
Other specialty	105 (5.8)	63 (4.9)	42 (8.2)	0.134	0.000
Unknown or not recorded	85 (4.7)	75 (5.8)	10 (1.9)	0.200	0.008
Operator training stage, No. (%)					
Resident, first year	885 (48.9)	675 (52.1)	210 (40.9)	0.227	0.015
Resident, second year	489 (27.0)	329 (25.4)	160 (31.1)	0.128	0.013
Resident, third year	78 (4.3)	61 (4.7)	17 (3.3)	0.071	0.039
Resident, fourth year	1 (0.1)	0	1 (0.2)	0.062	0.063
Attending physician	213 (11.8)	133 (10.3)	80 (15.6)	0.159	0.096
Medical student	76 (4.2)	46 (3.5)	30 (5.8)	0.108	0.033
Other operator	4 (0.2)	3 (0.2)	1 (0.2)	0.008	0.007
Unknown or not recorded	64 (3.5)	49 (3.8)	15 (2.9)	0.048	0.065
Operator age, median (IQR), y	27 (25-29)	27 (25-29)	27 (25-29)	0.062	0.002
Operator experience, median (IQR), y	2 (1-3)	2 (1-3)	2 (1-3)	0.066	0.010

Abbreviations: ASMD, absolute standardized mean difference; BMI, body mass index (calculated as weight in kilograms divided by height in meters squared); DL, direct laryngoscopy; ICU, intensive care unit; SOFA, Sequential Organ Failure Assessment; Spo_2_, peripheral oxygen saturation; VL, videolaryngoscope.

^a^ Absolute standardized mean differences between patients administered ketamine or etomidate in the unweighted and inverse propensity weighted cohorts.

### Primary Outcome

At 28 days, mortality was 60.5% (95% CI, 57.2%-63.8%) in the etomidate group and 54.4% (95% CI, 45.0%-63.9%) in the ketamine group (Table 1). In the primary analysis, patients who received etomidate had a higher risk of 28-day mortality compared with those who received ketamine (weighted RR, 1.14 [95% CI, 1.03-1.27]; *P* = .01; weighted RD, 7.6% [95% CI, 2.0%-13.3%]; *P* = .008). The Figure presents the weighted cumulative incidence of mortality over 28 days.

**Figure.**
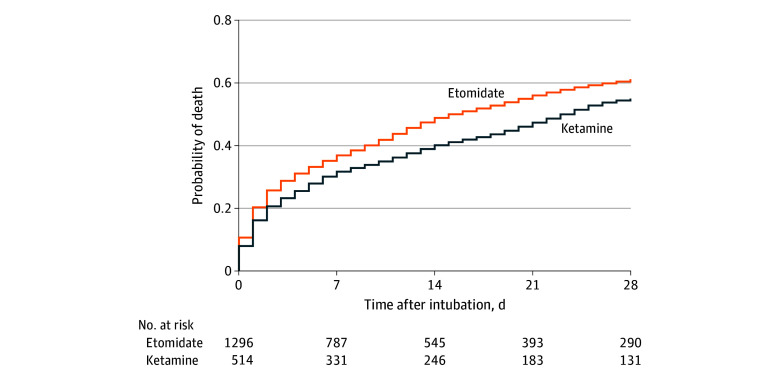
Inverse Propensity Weighted Kaplan-Meier Cumulative Incidence of Death Among Patients Administered Etomidate or Ketamine During Rapid-Sequence Intubation in the Emergency Department

### Secondary Outcomes

Table 2 summarizes secondary outcomes. At 7 days after intubation, the weighted risk of death was 35.2% (95% CI, 32.0%-38.3%) in the etomidate group and 30.1% (95% CI, 23.5%-36.7%) in the ketamine group, corresponding to an RR of 1.19 (95% CI, 1.04-1.35; *P* = .009) and an RD of 5.7% (95% CI, 1.6%-9.9%; *P* = .007).

**Table 2.** Association Between Administration of Etomidate or Ketamine for Rapid-Sequence Intubation and Patient Mortality After Intubation at 7 and 28 Days

Patient outcome	Weighted cumulative incidence or prevalence, % (95% CI)		Weighted risk ratio (95% CI)^a^	*P* value	Weighted risk difference (95% CI)^a^	*P* value
	Ketamine (reference)	Etomidate				
7-d Mortality	30.1 (23.5 to 36.7)	35.2 (32.0 to 38.3)	1.19 (1.04 to 1.35)	.009	0.057 (0.016 to 0.099)	.007
28-d Mortality	54.4 (45.0 to 63.9)	60.5 (57.2 to 63.8)	1.14 (1.03 to 1.27)	.01	0.076 (0.020 to 0.133)	.008
First-attempt intubation success	76.4 (72.6 to 80.2)	74.2 (71.8 to 76.6)	0.97 (0.92 to 1.03)	.34	−0.022 (−0.068 to 0.023)	.32
Any major adverse event	30.8 (26.5 to 35.2)	31.8 (29.3 to 34.3)	1.07 (0.92 to 1.24)	.36	0.012 (−0.037 to 0.061)	.63
New hemodynamic instability within 30 min after intubation	24.2 (20.4 to 28.0)	18.9 (16.7 to 21.0)	0.78 (0.64 to 0.95)	.01	−0.053 (−0.096 to −0.010)	.02
Severe hypoxemia	10.9 (8.2 to 13.7)	13.8 (11.9 to 15.6)	1.26 (0.94 to 1.67)	.12	0.028 (−0.005 to 0.061)	.10
Cardiac arrest	2.1 (0.8 to 3.3)	3.1 (2.1 to 4.0)	1.49 (0.75 to 2.95)	.25	0.010 (−0.006 to 0.026)	.21

^a^ All models were inverse propensity weighted.

Major adverse events occurred in 31.8% (95% CI, 29.3%-34.3%) of patients in the etomidate group and 30.8% (95% CI, 26.5%-35.2%) of patients in the ketamine group (RR, 1.07 [95% CI, 0.92-1.24]; RD, 1.2% [95% CI, −3.7% to 6.1%]; *P* = .63) (Table 2). New hemodynamic instability within 30 minutes after intubation was less frequent in the etomidate group compared with the ketamine group (18.9% [95% CI, 16.7%-21.0%] vs 24.2% [95% CI, 20.4%-28.0%]; RR, 0.78 [95% CI, 0.64-0.95]; RD, −5.3% [95% CI, −9.6% to −1.0%]; *P* = .02). Severe hypoxemia occurred in 13.8% (95% CI, 11.9%-15.6%) of patients in the etomidate group compared with 10.9% (95% CI, 8.2%-13.7%) of patients in the ketamine group (RR, 1.26 [95% CI, 0.94-1.67]; RD, 2.8% [95% CI, −0.5% to 6.1%]; *P* = .10). Cardiac arrest was observed in 3.1% (95% CI, 2.1%-4.0%) of patients in the etomidate group compared with 2.1% (95% CI, 0.8%-3.3%) of patients in the ketamine group (RR, 1.49 [95% CI, 0.75-2.95]; RD, 1.0% [95% CI, −0.6% to 2.6%]; *P* = .21).

The probability of first-attempt intubation success was similar between groups: 74.2% (95% CI, 71.8%-76.6%) in the etomidate group and 76.4% (95% CI, 72.6%-80.2%) in the ketamine group (RR, 0.97 [95% CI, 0.92-1.03]; RD, −2.2% [95% CI, −6.8% to 2.3%]; *P* = .32) (Table 2).

### Sensitivity Analyses

When adjusting for potential clustering by hospital through inclusion of a random intercept, etomidate remained associated with an increased risk of 28-day mortality compared with ketamine (RR, 1.24 [95% CI, 1.03-1.51]; RD, 7.7% [95% CI, 2.1%-13.4%]) (Table 3). Findings were similar when restricting the analysis to centers with at least 10 patients who received ketamine (RR, 1.16 [95% CI, 1.05-1.29]; RD, 8.8% [95% CI, 2.9%-14.7%]). The E-value for the point estimate (RR, 1.14 [95% CI, 1.03-1.27]) of 28-day mortality was 1.54 (E-value for the lower limit of the 95% CI, 1.21) (eFigure 2 in Supplement 1).

**Table 3.** Association Between Administration of Etomidate or Ketamine for Rapid-Sequence Intubation and Patient Mortality After Intubation at 7 and 28 Days Under Different Modeling Assumptions

Patient outcome	Weighted cumulative incidence or prevalence, % (95% CI)		Weighted risk ratio (95% CI)^a^	*P* value	Weighted risk difference (95% CI)^a^	*P* value
	Ketamine (reference)	Etomidate				
**Primary analysis**						
7-d Mortality	30.1 (23.5 to 36.7)	35.2 (32.0 to 38.3)	1.19 (1.04 to 1.35)	.009	0.057 (0.016 to 0.099)	.007
28-d Mortality	54.4 (45.0 to 63.9)	60.5 (57.2 to 63.8)	1.14 (1.03 to 1.27)	.01	0.076 (0.020 to 0.133)	.008
**Including random effect for hospital**						
7-d Mortality	30.1 (23.5 to 36.7)	35.2 (32.0 to 38.3)	1.27 (1.08 to 1.49)	.003	0.058 (0.016 to 0.100)	.006
28-d Mortality	54.4 (45.0 to 63.9)	60.5 (57.2 to 63.8)	1.24 (1.03 to 1.51)	.03	0.077 (0.021 to 0.134)	.007
**Excluding hospitals with <10 patients receiving ketamine**						
7-d Mortality	30.1 (23.4 to 36.8)	35.8 (32.4 to 39.2)	1.22 (1.06 to 1.39)	.004	0.066 (0.022 to 0.110)	.003
28-d Mortality	54.5 (44.8 to 64.3)	62.0 (58.8 to 65.2)	1.16 (1.05 to 1.29)	.005	0.088 (0.029 to 0.147)	.004

^a^ All models were inverse propensity weighted.

## Discussion

In this emulated target trial of 1810 critically ill adults undergoing RSI in the ED, etomidate as the induction agent was associated with increased mortality at both 7 and 28 days compared with ketamine. This association remained robust across sensitivity analyses, even though patients who received etomidate experienced fewer episodes of peri-intubation hemodynamic instability. Our results therefore reinforce the signal of potential harm reported in earlier observational studies and meta-analyses examining etomidate use in critically ill populations.

In the KETASED (Ketamine Versus Etomidate During Rapid Sequence Intubation) trial, ketamine was shown to be an alternative to etomidate as the induction agent in emergency intubations.^19^ In that trial, the hazard ratio for 28-day survival was 1.2 (95% CI, 0.9-1.6). Subsequently, the EvK (etomidate versus ketamine) trial showed higher 7-day mortality with etomidate; by 28 days the difference was not significant despite survival rates of 64.1% with etomidate vs 68.8% with ketamine (RD, 4.7%).^8^ In a large database study, Wunsch et al^20^ reported higher hospital mortality with etomidate vs ketamine (21.6% vs 18.7%; adjusted odds ratio, 1.28; 95% CI, 1.21-1.34). Our results expand on these findings and add precision to effect estimates. Our results align and add precision, with an RD of 7.6% and an RR of 1.14. The relative effect mirrors prior trials, with tighter confidence intervals due to the larger sample. The larger absolute difference likely reflects higher baseline mortality in our cohort compared with lower-mortality settings (eg, North America).

Unmeasured confounding may be a possible explanation for our results. A moderate-strength unmeasured confounder could attenuate the observed results, as the E-value for our primary outcome was 1.54 on the RR scale. Nevertheless, even a strong confounder such as lactate (which we did not routinely measure to include in our analysis) has an adjusted odds ratio for mortality (in our setting) of 1.11.^21^ This suggests that unmeasured confounding is unlikely to meaningfully change the final interpretation of our results.

A bayesian meta-analysis by Koroki et al^22^ estimated an 83% posterior probability that ketamine lowers mortality vs etomidate (RR, 0.93), but this signal was attenuated with conventional random-effects methods.^22,23^ By contrast, a meta-analysis of 11 randomized clinical trials (n = 2704) found no difference between etomidate and ketamine in mortality at the longest follow-up (RR, 1.07; 95% CI, 0.95-1.21).^24^ A recent meta-analysis of 7 randomized clinical trials (n = 2384) reported higher hemodynamic instability with ketamine (RR, 1.29; 95% CI, 1.07-1.57), lower adrenal suppression (RR, 0.54; 95% CI, 0.45-0.66), and reduced need for continuous vasopressors (RR, 0.75; 95% CI, 0.57-1.00).^9^ Overall, evidence suggests a trade-off: more early instability with ketamine, but preserved adrenal function and potentially less dependence on vasopressors.^4,25^

Etomidate’s apparent harm, despite a lower rate of peri-intubation hemodynamic instability (24.2% [ketamine] vs 18.9% [etomidate]), suggests competing effects. Although chosen for cardiovascular stability, etomidate transiently suppresses adrenal steroidogenesis and may blunt the stress response in sepsis or shock.^26^ In KETASED, etomidate increased adrenal insufficiency and organ dysfunction vs ketamine,^19^ and in the CORTICUS (Corticosteroid Therapy of Septic Shock) study, etomidate-associated adrenal suppression persisted regardless of hydrocortisone use.^27^ These findings conflict with the 2023 Society of Critical Care Medicine guidelines, which recommended either agent for RSI.^28^ Differences in analytic methods and case mix likely explain the discrepancy. In our cohort, patients receiving ketamine were sicker at baseline (higher shock index and more preintubation vasopressor use), which would bias against ketamine, yet etomidate remained associated with higher in-hospital mortality.

Together with a recent multicenter observational study^20^ and a systematic review,^22^ our findings question the routine use of etomidate for induction in critically ill adults undergoing intubation. This interpretation should not be extended to other indications (eg, procedural sedation in noncritically ill patients). We did not assess longer-term adverse effects of ketamine, which warrant study.^29,30^ A large randomized clinical trial is under way (NCT05277896). When available, an updated synthesis integrating randomized and rigorously adjusted observational data should inform recommendations. Marked interhospital variation in ketamine use underscores the need for standardized, evidence-based guidance.

### Strengths and Limitations

This study as some strengths, including a large, multicenter cohort of critically ill adults undergoing emergency intubation in Brazil, supporting generalizability across diverse settings; use of a target-trial emulation framework to strengthen causal inference; and a sample size sufficient to yield more precise effect estimates and extend prior work. However, the study also has limitations. Clinicians may have selected ketamine or etomidate based on factors not fully captured. Patients receiving ketamine were sicker at baseline (higher shock index and more preintubation vasopressor use), which would bias against ketamine; however, after IPTW adjustment and sensitivity analyses, etomidate remained associated with higher in-hospital mortality. Residual and unmeasured confounding may persist and could attenuate the observed associations. We also did not collect validated neurologic or functional outcomes at discharge or follow-up, precluding assessment of disability-free survival.^31^ Finally, 302 patients were intubated at urgent care or emergency units without on-site intensive care units and transferred elsewhere; 28-day outcomes were not captured for these cases. This logistics-driven exclusion may introduce selection bias and limit generalizability, particularly to centers with on-site intensive care units. Although results were consistent in sensitivity analyses, including models with hospital random effects, residual bias from transfers cannot be excluded.

## Conclusions

In this cohort study using a target trial emulation of critically ill adults undergoing RSI, use of etomidate was associated with higher in-hospital mortality at 7 and 28 days compared with ketamine, although etomidate was also associated with a lower incidence of peri-intubation hemodynamic instability. These findings highlight the need for definitive randomized clinical trials to compare the 2 agents.

## Notes

Supplement 1. **eAppendix.** Participating Centers **eFigure 1**. Patient Inclusion Flow Diagram **eFigure 2.** Sensitivity Analysis (E-Value) for Unmeasured Confounding for the Primary Outcome (28-Day In-Hospital Mortality) Analysis **eFigure 3.** Distribution of Propensity Scores for the Administration of Ketamine Versus Etomidate, Averaged Across All Imputed Datasets **eTable 1.** Target Trial Framework and Emulated Design **eTable 2.** Hospital Usage of Etomidate and Ketamine for Rapid Sequence Intubation **eTable 3.** Summary of Propensity Scores Among Patients Treated With Ketamine and Etomidate

Supplement 2. **Nonauthor Collaborators.** Brazilian Airway Registry Cooperation (BARCO) Group

Supplement 3.
Data Sharing Statement
