# Supplementary material for: Ketamine, Etomidate, and Mortality in Emergency Department Intubations
Source: JAMA Netw Open. 2025 Dec 15;8(12):e2548060. doi: 10.1001/jamanetworkopen.2025.48060 (PMC12706683; doi:10.1001/jamanetworkopen.2025.48060)
Supplement: Supplement 1. — eAppendix. Participating Centers eFigure 1. Patient Inclusion Flow Diagram eFigure 2. Sensitivity Analysis (E-Value) for Unmeasured Confounding for the Primary Outcome (28-Day In-Hospital Mortality) Analysis eFigure 3. Distribution of Propensity Scores for the Administration of Ketamine Versus Etomidate, Averaged Across All Imputed Datasets eTable 1. Target Trial Framework and Emulated Design eTable 2. Hospital Usage of Etomidate and Ketamine for Rapid Sequence Intubation eTable 3. Summary of Propensity Scores Among Patients Treated With Ketamine and Etomidate [file jamanetwopen-e2548060-s001.pdf]

Supplementary Online Content

Maia IWA, Decker SRR, Oliveira J e Silva L, et al. Ketamine, etomidate, and mortality in emergency department intubations. *JAMA Netw Open.* 2025;8(12):e2548060. doi:10.1001/jamanetworkopen.2025.48060

eAppendix. Participating Centers
eFigure 1. Patient Inclusion Flow Diagram
eFigure 2. Sensitivity Analysis (E-Value) for Unmeasured Confounding for the Primary Outcome (28-Day In-Hospital Mortality) Analysis
eFigure 3. Distribution of Propensity Scores for the Administration of Ketamine Versus Etomidate, Averaged Across All Imputed Datasets
eTable 1. Target Trial Framework and Emulated Design
eTable 2. Hospital Usage of Etomidate and Ketamine for Rapid Sequence Intubation
eTable 3. Summary of Propensity Scores Among Patients Treated With Ketamine and Etomidate

This supplementary material has been provided by the authors to give readers additional information about their work.

© 2025 Maia IWA et al. *JAMA Network Open.*

eAppendix. Participating Centers

The network included 18 emergency care centers spanning 6 Brazilian states (São Paulo, Rio Grande do Sul, Santa Catarina, Minas Gerais, Ceará, and Mato Grosso) and 13 municipalities. Sites included 17 hospital-based emergency departments, most affiliated with public universities or large referral hospitals, and one municipal urgent care unit (UPA Lajeado). Geographic coverage spanned the South, Southeast, Northeast, and Central-West regions of Brazil. The participating centers were:

Department of Emergency Medicine, Hospital das Clínicas da Faculdade de Medicina da Universidade de São Paulo (HCFMUSP), São Paulo, São Paulo, Brazil

Department of Emergency Medicine, Hospital de Clínicas de Porto Alegre (HCPA), Porto Alegre, Rio Grande do Sul, Brazil

Department of Emergency Medicine, Hospital Nossa Senhora da Conceição (Grupo Hospitalar Conceição), Porto Alegre, Rio Grande do Sul, Brazil

Department of Emergency Medicine, Hospital de Pronto Socorro de Porto Alegre (HPS), Porto Alegre, Rio Grande do Sul, Brazil

Department of Emergency Medicine, Hospital São Lucas da Pontifícia Universidade Católica do Rio Grande do Sul (PUCRS), Porto Alegre, Rio Grande do Sul, Brazil

Department of Emergency Medicine, Hospital Santa Cruz, Santa Cruz do Sul, Rio Grande do Sul, Brazil

Department of Emergency Medicine, Hospital Bruno Born, Lajeado, Rio Grande do Sul, Brazil

Unidade de Pronto Atendimento de Lajeado (UPA Lajeado), Lajeado, Rio Grande do Sul, Brazil

Department of Emergency Medicine, Hospital Regional Alto Vale, Rio do Sul, Santa Catarina, Brazil

Department of Emergency Medicine, Hospital Regional de São José Dr. Homero de Miranda Gomes, São José, Santa Catarina, Brazil

Department of Emergency Medicine, Hospital Metropolitano Odilon Behrens, Belo Horizonte, Minas Gerais, Brazil

Department of Emergency Medicine, Hospital Geral de Fortaleza, Fortaleza, Ceará, Brazil

Department of Emergency Medicine, Hospital de Messejana Dr. Carlos Alberto Studart Gomes, Fortaleza, Ceará, Brazil

Department of Emergency Medicine, Hospital Santo Antônio, Sinop, Mato Grosso, Brazil

Department of Emergency Medicine, Hospital Augusto de Oliveira Camargo (HAOC), Indaiatuba, São Paulo, Brazil

Department of Emergency Medicine, Faculdade de Medicina de Ribeirão Preto, University of São Paulo (FMRP-USP), Ribeirão Preto, São Paulo, Brazil

Department of Emergency Medicine, Universidade Estadual Paulista (UNESP), Botucatu, São Paulo, Brazil

Department of Emergency Medicine, Hospital das Clínicas da Faculdade de Medicina de Marília (FAMEMA), Marília, São Paulo, Brazil

© 2025 Maia IWA et al. *JAMA Network Open.*

eFigure 1. Patient Inclusion Flow Diagram

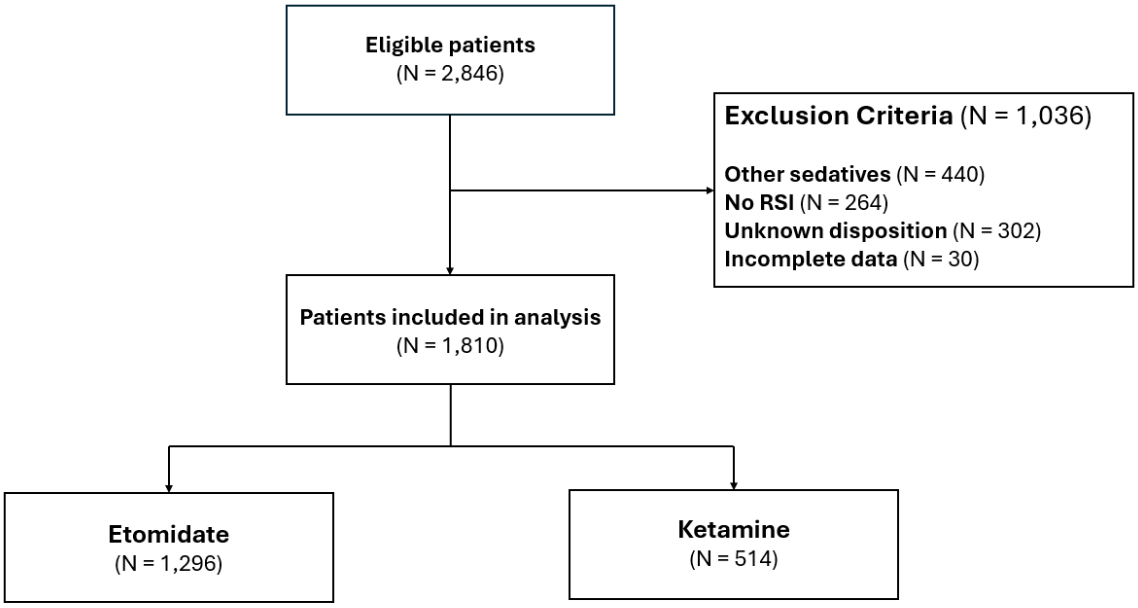

RSI - Rapid Sequence Intubation

© 2025 Maia IWA et al. *JAMA Network Open.*

eFigure 2. Sensitivity Analysis (E-Value) for Unmeasured Confounding for the Primary Outcome (28-Day In-Hospital Mortality) Analysis

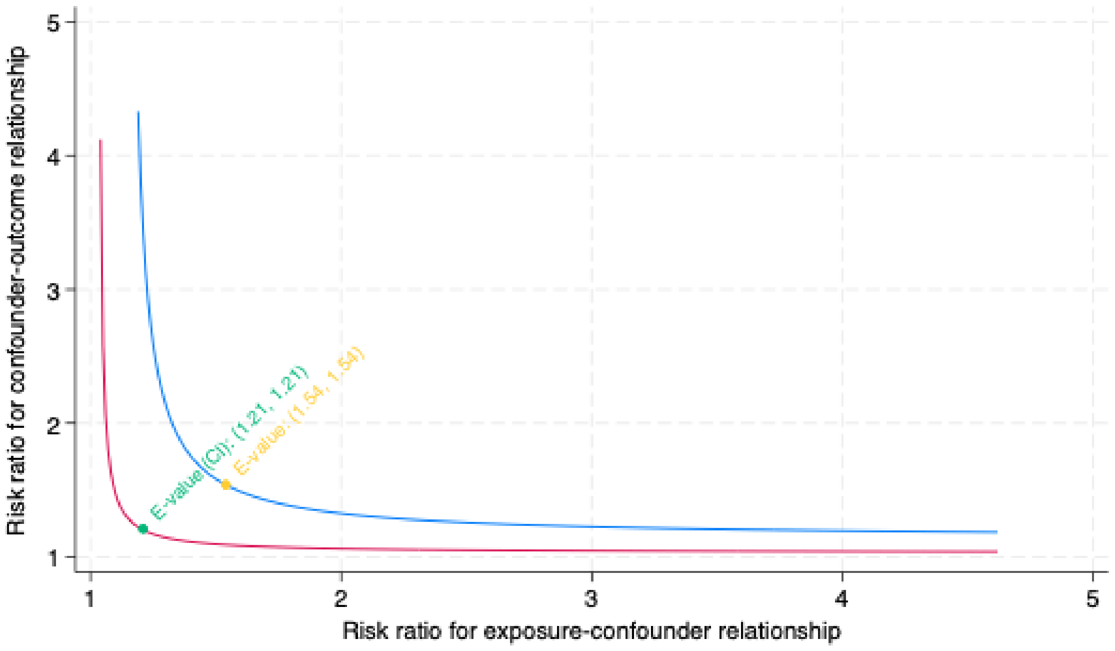

© 2025 Maia IWA et al. *JAMA Network Open.*

eFigure 3. Distribution of Propensity Scores for the Administration of Ketamine Versus Etomidate, Averaged Across All Imputed Datasets

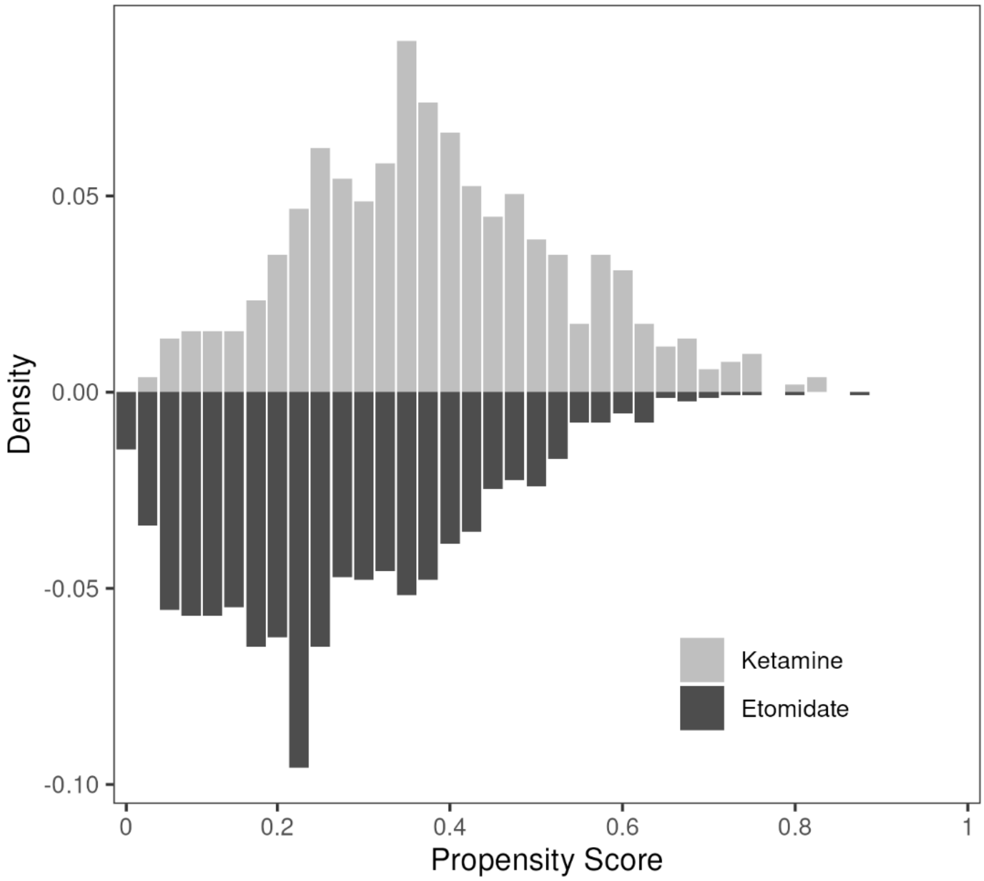

© 2025 Maia IWA et al. *JAMA Network Open.*

eTable 1. Target Trial Framework and Emulated Design

Component	Target Trial	Emulated Design
**Causal Question**	Among critically ill adults undergoing RSI, does etomidate vs. ketamine increase 28-day in-hospital mortality?	Same causal question applied to prospectively collected data.
**Eligibility Criteria**	Adults aged ≥18 years undergoing RSI in the emergency department; excluded patients with pre-intubation cardiac arrest.	BARCO participants meeting the same criteria; additionally excluded those transferred immediately post-intubation
**Treatment Strategies**	Single bolus etomidate vs. ketamine.	Receipt of etomidate or ketamine as the sole induction agent recorded in the registry; both followed by neuromuscular blockade. Doses not standardized.
**Treatment Assignment**	Individual randomization at induction	Observational treatment assignment at a well-defined time point (induction). Emulated randomization via inverse probability of treatment weighting using propensity scores.
**Follow-up**	From induction through hospital discharge, death, or 28 days post-intubation, whichever occurred first.	Same follow-up period applied in the registry. Patients without definitive outcome data were excluded.
**Causal Estimand**	Per-protocol effect and intention-to-treat effect	Per-protocol effect, assuming conditional exchangeability

© 2025 Maia IWA et al. *JAMA Network Open.*

**Outcome Definition**	In-hospital all-cause mortality at 28 days (primary); 7-day mortality and major adverse events as secondary outcomes.	Identical outcome definitions applied using prospectively collected BARCO registry data.

RSI - Rapid Sequence Intubation

© 2025 Maia IWA et al. *JAMA Network Open.*

eTable 2. Hospital Usage of Etomidate and Ketamine for Rapid Sequence Intubation

**Hospital**	**Number of Patients**	**Etomidate**, n (%)	**Ketamine**, n (%)	**28-Day Mortality**, n (%)
Center 1	3	3 (100%)	0 (0%)	0 (0%)
Center 2	50	49 (98%)	1 (2%)	22 (44%)
Center 3	61	58 (95%)	3 (5%)	26 (43%)
Center 4	21	20 (95%)	1 (5%)	13 (62%)
Center 5	104	94 (90%)	10 (10%)	63 (61%)
Center 6	83	74 (89%)	9 (11%)	39 (47%)
Center 7	303	263 (87%)	40 (13%)	188 (62%)
Center 8	23	20 (87%)	3 (13%)	9 (39%)
Center 9	171	147 (86%)	24 (14%)	88 (51%)
Center 10	47	39 (83%)	8 (17%)	29 (62%)
Center 11	42	30 (71%)	12 (29%)	22 (52%)
Center 12	265	179 (68%)	86 (32%)	109 (41%)
Center 13	269	166 (62%)	103 (38%)	136 (51%)
Center 14	24	14 (58%)	10 (42%)	17 (71%)
Center 15	35	19 (54%)	16 (46%)	31 (89%)
Center 16	85	43 (51%)	42 (49%)	48 (56%)
Center 17	122	52 (43%)	70 (57%)	57 (47%)
Center 18	102	26 (25%)	76 (75%)	72 (71%)
**Overall**	**1810**	**1296 (71.6%)**	**514 (28.4%)**	**969 (53.5%)**

© 2025 Maia IWA et al. *JAMA Network Open.*

eTable 3. Summary of Propensity Scores Among Patients Treated With Ketamine and Etomidate

	Percentile						
	1	10	25	50	75	90	99
**Ketamine**	0.0685	0.1868	0.2602	0.3552	0.4638	0.5771	0.7343
**Etomidate**	0.0225	0.0715	0.1419	0.2254	0.3495	0.4499	0.6147

© 2025 Maia IWA et al. *JAMA Network Open.*
