# Supplementary material for: Ketamine, Etomidate, and Mortality in Emergency Department Intubations
Source: JAMA Netw Open. 2025 Dec 15;8(12):e2548060. doi: 10.1001/jamanetworkopen.2025.48060 (PMC12706683; doi:10.1001/jamanetworkopen.2025.48060)
Supplement: Supplement 2. — Nonauthor Collaborators. Brazilian Airway Registry Cooperation (BARCO) Group [file jamanetwopen-e2548060-s002.pdf]

Supplemental Online Content: Nonauthor Collaborators
*First name, last name, and suffix (if applicable) are required and will appear in PubMed.

*Group Name(s): Brazilian Airway Registry Cooperation (BARCO) group							
***First Name and Middle Initial(s)**	***Last Name**	***Suffix (eg, Jr, III)**	Academic Degrees	Institution	Location (city, state/province, country)	Role or Contribution, eg, chair, principal investigator	Group (if more than 1 Group listed in the byline) and/or Subgroup (eg, Steering Committee)
Carolline	Alaia Colin		MD	Hospital Augusto de Oliveira Camargo	Indaiatuba, Sao Paulo, Brazil	Data collection	
Andrey Caique Jorge	da Silva		MD	Hospital Augusto de Oliveira Camargo	Indaiatuba, Sao Paulo, Brazil	Data collection	
Andre	Dos Santos Cleto		MD	Hospital Augusto de Oliveira Camargo	Indaiatuba, Sao Paulo, Brazil	Data collection	
Gabriele	Escocia Marinho		MD	Hospital Augusto de Oliveira Camargo	Indaiatuba, Sao Paulo, Brazil	Data collection	
Vanessa	Giffoni		MD	Hospital Augusto de Oliveira Camargo	Indaiatuba, Sao Paulo, Brazil	Data collection	
Hilana	K Zenly		MD	Hospital Augusto de Oliveira Camargo	Indaiatuba, Sao Paulo, Brazil	Data collection	
Guilherme	Moreno Navarro		MD	Hospital Augusto de Oliveira Camargo	Indaiatuba, Sao Paulo, Brazil	Data collection	
Paola Horanna	Rodrigues de Freitas		MD	Hospital Augusto de Oliveira Camargo	Indaiatuba, Sao Paulo, Brazil	Data collection	
Hiram	Silva		MD	Hospital Augusto de Oliveira Camargo	Indaiatuba, Sao Paulo, Brazil	Data collection	
Kaue	Teixeira Lima		MD	Hospital Augusto de Oliveira Camargo	Indaiatuba, Sao Paulo, Brazil	Data collection	
Gabriela	Zanon		MD	Hospital Augusto de Oliveira Camargo	Indaiatuba, Sao Paulo, Brazil	Data collection	
Samara	Pinheiro De Souza		MD	Hospital Augusto de Oliveira Camargo	Indaiatuba, Sao Paulo, Brazil	Regional Principal Investigator	
Larissa Ferreira Alves da Silva	Alves da Silva		MD	Hospital Bruno Born	Lajeado, Rio Grande do Sul, Brazil	Data collection	
Cristian	Amaral Pereira		MD	Hospital Bruno Born	Lajeado, Rio Grande do Sul, Brazil	Data collection	

Supplemental Online Content: Nonauthor Collaborators
*First name, last name, and suffix (if applicable) are required and will appear in PubMed.

*First Name and Middle Initial(s)	*Last Name	*Suffix (eg, Jr, III)	Academic Degrees	Institution	Location (city, state/province, country)	Role or Contribution, eg, chair, principal investigator	Group (if more than 1 Group listed in the byline) and/or Subgroup (eg, Steering Committee)
Cirio Osvaldo	Auler		MS	Hospital Bruno Born	Lajeado, Rio Grande do Sul, Brazil	Data collection	
Bruna	Brignoli Bernardino		MD	Hospital Bruno Born	Lajeado, Rio Grande do Sul, Brazil	Data collection	
Bruna	Bruna Zagonel		MD	Hospital Bruno Born	Lajeado, Rio Grande do Sul, Brazil	Data collection	
Eduarda	Capra Bertolin		MS	Hospital Bruno Born	Lajeado, Rio Grande do Sul, Brazil	Data collection	
Yuri	Carlotto Ramires		MD	Hospital Bruno Born	Lajeado, Rio Grande do Sul, Brazil	Data collection	
Sibele	Catarina Bernardi Jacob		MD	Hospital Bruno Born	Lajeado, Rio Grande do Sul, Brazil	Data collection	
Vinicius	cius Sartor Henrique		MD	Hospital Bruno Born	Lajeado, Rio Grande do Sul, Brazil	Data collection	
Cleiton	Cordeiro Prola		MS	Hospital Bruno Born	Lajeado, Rio Grande do Sul, Brazil	Data collection	
Rita	de Souza Melo		MD	Hospital Bruno Born	Lajeado, Rio Grande do Sul, Brazil	Data collection	
Andrea	de Vargas Tomelero		MD	Hospital Bruno Born	Lajeado, Rio Grande do Sul, Brazil	Data collection	
Eduardo	Eduardo Scheffel		MS	Hospital Bruno Born	Lajeado, Rio Grande do Sul, Brazil	Data collection	
Fernando	Fernando Morgado		MS	Hospital Bruno Born	Lajeado, Rio Grande do Sul, Brazil	Data collection	
Marjurye	Gross Ramos Pereira		MD	Hospital Bruno Born	Lajeado, Rio Grande do Sul, Brazil	Data collection	
Eduardo Henrique	Guadagnin		MS	Hospital Bruno Born	Lajeado, Rio Grande do Sul, Brazil	Data collection	
Shanna	Habner		MS	Hospital Bruno Born	Lajeado, Rio Grande do Sul, Brazil	Data collection	

Supplemental Online Content: Nonauthor Collaborators
*First name, last name, and suffix (if applicable) are required and will appear in PubMed.

*First Name and Middle Initial(s)	*Last Name	*Suffix (eg, Jr, III)	Academic Degrees	Institution	Location (city, state/province, country)	Role or Contribution, eg, chair, principal investigator	Group (if more than 1 Group listed in the byline) and/or Subgroup (eg, Steering Committee)
Helena Kilian	Helena Kilian		MS	Hospital Bruno Born	Lajeado, Rio Grande do Sul, Brazil	Data collection	
Eduardo	Jaeger Mensch		MD	Hospital Bruno Born	Lajeado, Rio Grande do Sul, Brazil	Data collection	
Lucas Oliveira Junqueira e Silva	Junqueira e Silva		MD	Hospital Bruno Born	Lajeado, Rio Grande do Sul, Brazil	Data collection	
Antonio Carlos	Klug Cogo		MD	Hospital Bruno Born	Lajeado, Rio Grande do Sul, Brazil	Data collection	
Natascha	Kokay Nepomuceno		MD	Hospital Bruno Born	Lajeado, Rio Grande do Sul, Brazil	Data collection	
Ana Luisa	Lanius		MD	Hospital Bruno Born	Lajeado, Rio Grande do Sul, Brazil	Data collection	
Lara Faria	Lara Faria		MS	Hospital Bruno Born	Lajeado, Rio Grande do Sul, Brazil	Data collection	
Bruna	Lavagnini		MD	Hospital Bruno Born	Lajeado, Rio Grande do Sul, Brazil	Data collection	
Gustavo Lavall Dill	Lavall Dill		MS	Hospital Bruno Born	Lajeado, Rio Grande do Sul, Brazil	Data collection	
Paulo Victor	Lopes		MD	Hospital Bruno Born	Lajeado, Rio Grande do Sul, Brazil	Data collection	
Luiz Artur	Nunes Ribeiro		MS	Hospital Bruno Born	Lajeado, Rio Grande do Sul, Brazil	Data collection	
Alvaro	Ortiga Maciel		MS	Hospital Bruno Born	Lajeado, Rio Grande do Sul, Brazil	Data collection	
Mariana	Pessini		MD	Hospital Bruno Born	Lajeado, Rio Grande do Sul, Brazil	Data collection	
Carolina Antonia	Pietrobon		MS	Hospital Bruno Born	Lajeado, Rio Grande do Sul, Brazil	Data collection	
Lucas Pires Freitas	Pires Freitas		MD	Hospital Bruno Born	Lajeado, Rio Grande do Sul, Brazil	Data collection	

Supplemental Online Content: Nonauthor Collaborators
*First name, last name, and suffix (if applicable) are required and will appear in PubMed.

*First Name and Middle Initial(s)	*Last Name	*Suffix (eg, Jr, III)	Academic Degrees	Institution	Location (city, state/province, country)	Role or Contribution, eg, chair, principal investigator	Group (if more than 1 Group listed in the byline) and/or Subgroup (eg, Steering Committee)
Morgana	Pizzolatti Marins		MD	Hospital Bruno Born	Lajeado, Rio Grande do Sul, Brazil	Data collection	
Ana Leticia	Pizzutti		MS	Hospital Bruno Born	Lajeado, Rio Grande do Sul, Brazil	Data collection	
Cleiton	Prola		MS	Hospital Bruno Born	Lajeado, Rio Grande do Sul, Brazil	Data collection	
Lucas	Rodrigues De Oliveira		MD	Hospital Bruno Born	Lajeado, Rio Grande do Sul, Brazil	Data collection	
Vinicius	S. Castro		MD	Hospital Bruno Born	Lajeado, Rio Grande do Sul, Brazil	Data collection	
Sabrina	Sabrina Favaretto		MS	Hospital Bruno Born	Lajeado, Rio Grande do Sul, Brazil	Data collection	
Alexandre	Schmidt		MS	Hospital Bruno Born	Lajeado, Rio Grande do Sul, Brazil	Data collection	
Luiz Augusto	Soares		MS	Hospital Bruno Born	Lajeado, Rio Grande do Sul, Brazil	Data collection	
Camila Zanco	Zanco		MD	Hospital Bruno Born	Lajeado, Rio Grande do Sul, Brazil	Data collection	
Liliane	de Oliveira Miranda		MD	Hospital das Clinicas da FM Ribeirao Preto	Ribeirao Preto, Sao Paulo, Brazil	Data collection	
Felipe	Barbosa Vallt		MD	Hospital das Clinicas da FM Ribeirao Preto	Ribeirao Preto, Sao Paulo, Brazil	Data collection	
Anna Cristina	Bertoldi Lemos Teodoro		MD	Hospital das Clinicas da FM Ribeirao Preto	Ribeirao Preto, Sao Paulo, Brazil	Data collection	
Isabella	Busnello Felipe		MD	Hospital das Clinicas da FM Ribeirao Preto	Ribeirao Preto, Sao Paulo, Brazil	Data collection	
Beatriz	Ceron Pretti		MD	Hospital das Clinicas da FM Ribeirao Preto	Ribeirao Preto, Sao Paulo, Brazil	Data collection	
Luis Felipe	Fucuta Figueira		MD	Hospital das Clinicas da FM Ribeirao Preto	Ribeirao Preto, Sao Paulo, Brazil	Data collection	

*First name, last name, and suffix (if applicable) are required and will appear in PubMed.

***First Name and Middle Initial(s)**	***Last Name**	***Suffix (eg, Jr, III)**	Academic Degrees	Institution	Location (city, state/province, country)	Role or Contribution, eg, chair, principal investigator	Group (if more than 1 Group listed in the byline) and/or Subgroup (eg, Steering Committee)
Marcelo	Guimaraes Coelho		MD	Hospital das Clinicas da FM Ribeirao Preto	Ribeirao Preto, Sao Paulo, Brazil	Data collection	
Aron	Hussid Ferreira		MD	Hospital das Clinicas da FM Ribeirao Preto	Ribeirao Preto, Sao Paulo, Brazil	Data collection	
Stella	Lage Vieira Abrantes dos Santos		MD	Hospital das Clinicas da FM Ribeirao Preto	Ribeirao Preto, Sao Paulo, Brazil	Data collection	
Gabriel	Queda Toledo		MD	Hospital das Clinicas da FM Ribeirao Preto	Ribeirao Preto, Sao Paulo, Brazil	Data collection	
Arthur	Reimann Oliveira		MD	Hospital das Clinicas da FM Ribeirao Preto	Ribeirao Preto, Sao Paulo, Brazil	Data collection	
Gabriela	Simões Belote		MD	Hospital das Clinicas da FM Ribeirao Preto	Ribeirao Preto, Sao Paulo, Brazil	Data collection	
Ana Caroline	Soares Costa		MD	Hospital das Clinicas da FM Ribeirao Preto	Ribeirao Preto, Sao Paulo, Brazil	Data collection	
Erick	Sobral Porto		MD	Hospital das Clinicas da FM Ribeirao Preto	Ribeirao Preto, Sao Paulo, Brazil	Data collection	
Leonardo	Soriano		MD	Hospital das Clinicas da FM Ribeirao Preto	Ribeirao Preto, Sao Paulo, Brazil	Data collection	
Valeria	Takeuchi Okino		MD	Hospital das Clinicas da FM Ribeirao Preto	Ribeirao Preto, Sao Paulo, Brazil	Data collection	
Rubens	Yoshinori Kai		MD	Hospital das Clinicas da FM Ribeirao Preto	Ribeirao Preto, Sao Paulo, Brazil	Regional Case Manager	
Renato	Araújo de Melo		MD	Hospital das Clinicas de Marilia	Marilia, Sao Paulo, Brazil	Data collection	
Roger	Baldi Castro		MD	Hospital das Clinicas de Marilia	Marilia, Sao Paulo, Brazil	Data collection	
Sheylla	Barbosa Rodacki Nunes		MS	Hospital das Clinicas de Marilia	Marilia, Sao Paulo, Brazil	Data collection	
Laura	Batista Carmo Silva		MD	Hospital das Clinicas de Marilia	Marilia, Sao Paulo, Brazil	Data collection	
Jordana	Blini Mariucio		MD	Hospital das Clinicas de Marilia	Marilia, Sao Paulo, Brazil	Data collection	

Supplemental Online Content: Nonauthor Collaborators
*First name, last name, and suffix (if applicable) are required and will appear in PubMed.

*First Name and Middle Initial(s)	*Last Name	*Suffix (eg, Jr, III)	Academic Degrees	Institution	Location (city, state/province, country)	Role or Contribution, eg, chair, principal investigator	Group (if more than 1 Group listed in the byline) and/or Subgroup (eg, Steering Committee)
Eduardo	Busso Magri		MS	Hospital das Clinicas de Marilia	Marilia, Sao Paulo, Brazil	Data collection	
Roque	Campos Alonge		Nurse	Hospital das Clinicas de Marilia	Marilia, Sao Paulo, Brazil	Data collection	
Tassiana	Colonese Gonçalves		MS	Hospital das Clinicas de Marilia	Marilia, Sao Paulo, Brazil	Data collection	
Giovanna	Cristine Martins Duarte		Other	Hospital das Clinicas de Marilia	Marilia, Sao Paulo, Brazil	Data collection	
Gabriella	de Andrade Moreira		MS	Hospital das Clinicas de Marilia	Marilia, Sao Paulo, Brazil	Data collection	
Luana	Demetrio Raia Ferranti		MS	Hospital das Clinicas de Marilia	Marilia, Sao Paulo, Brazil	Data collection	
Nathália	Desiderio		MS	Hospital das Clinicas de Marilia	Marilia, Sao Paulo, Brazil	Data collection	
Juliana	Donato e Castro		MS	Hospital das Clinicas de Marilia	Marilia, Sao Paulo, Brazil	Data collection	
Julia	Eico Nakamura		MD	Hospital das Clinicas de Marilia	Marilia, Sao Paulo, Brazil	Data collection	
Thaís	Eigler Sampedro		MS	Hospital das Clinicas de Marilia	Marilia, Sao Paulo, Brazil	Data collection	
Thiago	Ferreira de Almeida		Respiratory therapist	Hospital das Clinicas de Marilia	Marilia, Sao Paulo, Brazil	Data collection	
Rodrigo	Gazzola Cunha		MD	Hospital das Clinicas de Marilia	Marilia, Sao Paulo, Brazil	Data collection	
Sara	Julian Pelaquim		MD	Hospital das Clinicas de Marilia	Marilia, Sao Paulo, Brazil	Data collection	
Mariana	Marin Gasparini		MD	Hospital das Clinicas de Marilia	Marilia, Sao Paulo, Brazil	Data collection	
Giovana	Marineli Silva		Nurse	Hospital das Clinicas de Marilia	Marilia, Sao Paulo, Brazil	Data collection	
Paula	Martins		MD	Hospital das Clinicas de Marilia	Marilia, Sao Paulo, Brazil	Data collection	
Bruno	Martins da Silva		MS	Hospital das Clinicas de Marilia	Marilia, Sao Paulo, Brazil	Data collection	
Larissa	Monteiro		MD	Hospital das Clinicas de Marilia	Marilia, Sao Paulo, Brazil	Data collection	

*First name, last name, and suffix (if applicable) are required and will appear in PubMed.

*First Name and Middle Initial(s)	*Last Name	*Suffix (eg, Jr, III)	Academic Degrees	Institution	Location (city, state/province, country)	Role or Contribution, eg, chair, principal investigator	Group (if more than 1 Group listed in the byline) and/or Subgroup (eg, Steering Committee)
Mísia	Nogueira Altino Wang		MD	Hospital das Clinicas de Marilia	Marilia, Sao Paulo, Brazil	Data collection	
Napoleao	Nunes Teixeira Filho		MD	Hospital das Clinicas de Marilia	Marilia, Sao Paulo, Brazil	Data collection	
José Maria	Pereira da Silva		Respiratory therapist	Hospital das Clinicas de Marilia	Marilia, Sao Paulo, Brazil	Data collection	
Isabela	Prado Campanhã		MS	Hospital das Clinicas de Marilia	Marilia, Sao Paulo, Brazil	Data collection	
Gabriella	Rakanidis Machado		MS	Hospital das Clinicas de Marilia	Marilia, Sao Paulo, Brazil	Data collection	
Letícia	Ribeiro Alvetti		MD	Hospital das Clinicas de Marilia	Marilia, Sao Paulo, Brazil	Data collection	
Vívian	Rodrigues		Nurse	Hospital das Clinicas de Marilia	Marilia, Sao Paulo, Brazil	Data collection	
Lucia	Rosa Passaglia		MS	Hospital das Clinicas de Marilia	Marilia, Sao Paulo, Brazil	Data collection	
Nathalia	Rossi Lage		MS	Hospital das Clinicas de Marilia	Marilia, Sao Paulo, Brazil	Data collection	
Rodrigo	Seiji Yasue		MS	Hospital das Clinicas de Marilia	Marilia, Sao Paulo, Brazil	Data collection	
Luísa	Serpa Vale		MS	Hospital das Clinicas de Marilia	Marilia, Sao Paulo, Brazil	Data collection	
Karina	Shimada		MS	Hospital das Clinicas de Marilia	Marilia, Sao Paulo, Brazil	Data collection	
Renan	Shinkawa Cassini		MS	Hospital das Clinicas de Marilia	Marilia, Sao Paulo, Brazil	Data collection	
Wendel	Thales		Nurse	Hospital das Clinicas de Marilia	Marilia, Sao Paulo, Brazil	Data collection	
Miriã	Villar de Oliveira		MD	Hospital das Clinicas de Marilia	Marilia, Sao Paulo, Brazil	Data collection	
Tuffi	Zina Neto		MD	Hospital das Clinicas de Marilia	Marilia, Sao Paulo, Brazil	Data collection	
Dulcinéi	a de Souza Rodrigues dos Santos		Nurse	Hospital das Clinicas de Marilia	Marilia, Sao Paulo, Brazil	Data collection	
Rafael	Albanês pires		MS	Hospital das Clinicas de Marilia	Marilia, Sao Paulo, Brazil	Data collection	
Laís S	anches Aguera		MD	Hospital das Clinicas de Marilia	Marilia, Sao Paulo, Brazil	Data collection	
Laísy	Araujo Guimarães		MS	Hospital das Clinicas de Marilia	Marilia, Sao Paulo, Brazil	Data collection	

Supplemental Online Content: Nonauthor Collaborators
*First name, last name, and suffix (if applicable) are required and will appear in PubMed.

*First Name and Middle Initial(s)	*Last Name	*Suffix (eg, Jr, III)	Academic Degrees	Institution	Location (city, state/province, country)	Role or Contribution, eg, chair, principal investigator	Group (if more than 1 Group listed in the byline) and/or Subgroup (eg, Steering Committee)
Joyce	Berteli		MD	Hospital das Clinicas de Marilia	Marilia, Sao Paulo, Brazil	Data collection	
Beatriz	Bianchi Gonçalves da Silva		MS	Hospital das Clinicas de Marilia	Marilia, Sao Paulo, Brazil	Data collection	
Lucas	Castello Branco		MD	Hospital das Clinicas de Marilia	Marilia, Sao Paulo, Brazil	Data collection	
Vitória Maria	Chacon Martins		MD	Hospital das Clinicas de Marilia	Marilia, Sao Paulo, Brazil	Data collection	
Luisa	Cunha de Almeida Aguilar		MD	Hospital das Clinicas de Marilia	Marilia, Sao Paulo, Brazil	Data collection	
Lucas Eduardo	Da Silva		Nurse	Hospital das Clinicas de Marilia	Marilia, Sao Paulo, Brazil	Data collection	
Maria Eduarda	de Arêa Leão		MS	Hospital das Clinicas de Marilia	Marilia, Sao Paulo, Brazil	Data collection	
Beatriz Tobias	De Oliveira		MS	Hospital das Clinicas de Marilia	Marilia, Sao Paulo, Brazil	Data collection	
Lais	de Paiva Xavier		MS	Hospital das Clinicas de Marilia	Marilia, Sao Paulo, Brazil	Data collection	
Amauri	Doreto da Rocha Filho		Other	Hospital das Clinicas de Marilia	Marilia, Sao Paulo, Brazil	Data collection	
Cauana Caroline	Dos Santos		Nurse	Hospital das Clinicas de Marilia	Marilia, Sao Paulo, Brazil	Data collection	
Anna Clara	Ferro		MD	Hospital das Clinicas de Marilia	Marilia, Sao Paulo, Brazil	Data collection	
Maria Clara	Figueroa da Silva		MS	Hospital das Clinicas de Marilia	Marilia, Sao Paulo, Brazil	Data collection	
Lyse	Gil		MS	Hospital das Clinicas de Marilia	Marilia, Sao Paulo, Brazil	Data collection	
João Paulo	Gobbo Coimbra		MS	Hospital das Clinicas de Marilia	Marilia, Sao Paulo, Brazil	Data collection	
Thaís	Koga		Nurse	Hospital das Clinicas de Marilia	Marilia, Sao Paulo, Brazil	Data collection	
Haysam Youssef	Magalhães		MD	Hospital das Clinicas de Marilia	Marilia, Sao Paulo, Brazil	Data collection	
André Rosa	Moreira Lima		MD	Hospital das Clinicas de Marilia	Marilia, Sao Paulo, Brazil	Data collection	
Tatyelle	Oliveira dos Santos		MS	Hospital das Clinicas de Marilia	Marilia, Sao Paulo, Brazil	Data collection	
Isabela	Pilan Mendonça		MD	Hospital das Clinicas de Marilia	Marilia, Sao Paulo, Brazil	Data collection	

Supplemental Online Content: Nonauthor Collaborators
*First name, last name, and suffix (if applicable) are required and will appear in PubMed.

***First Name and Middle Initial(s)**	***Last Name**	***Suffix (eg, Jr, III)**	Academic Degrees	Institution	Location (city, state/province, country)	Role or Contribution, eg, chair, principal investigator	Group (if more than 1 Group listed in the byline) and/or Subgroup (eg, Steering Committee)
Juliana	Ribeiro Neves de Vasconcellos		MS	Hospital das Clinicas de Marilia	Marilia, Sao Paulo, Brazil	Data collection	
Maria Fernanda	Rosa Dezan		MD	Hospital das Clinicas de Marilia	Marilia, Sao Paulo, Brazil	Data collection	
José Luís	Simões Júnior		MD	Hospital das Clinicas de Marilia	Marilia, Sao Paulo, Brazil	Data collection	
Paula	Sylvana Martins da Silva		MD	Hospital das Clinicas de Marilia	Marilia, Sao Paulo, Brazil	Data collection	
Reginaldo	Trombini		Other	Hospital das Clinicas de Marilia	Marilia, Sao Paulo, Brazil	Data collection	
Luís Augusto	Veronezzi Savioli		MS	Hospital das Clinicas de Marilia	Marilia, Sao Paulo, Brazil	Data collection	
Ariadine Augusta	Maiante		MD	Hospital das Clinicas de Marilia	Marilia, Sao Paulo, Brazil	Regional Case Manager	
Bianca	Bertuzzi		MD	Hospital de Clinicas de Porto Alegre	Porto Alegre, Rio Grande do Sul, Brazil	Data collection	
Ariane	Coester		MD	Hospital de Clinicas de Porto Alegre	Porto Alegre, Rio Grande do Sul, Brazil	Data collection	
Pedro Henrique	de Almeida Francisco		MD	Hospital de Clinicas de Porto Alegre	Porto Alegre, Rio Grande do Sul, Brazil	Data collection	
João Vitor	de Camargo Zimmer Vilhena		MD	Hospital de Clinicas de Porto Alegre	Porto Alegre, Rio Grande do Sul, Brazil	Data collection	
Carolina	Fagundes Dall'Oglio		MD	Hospital de Clinicas de Porto Alegre	Porto Alegre, Rio Grande do Sul, Brazil	Data collection	
Rafael	Lima Mc Gregor Von Hellmann		MD	Hospital de Clinicas de Porto Alegre	Porto Alegre, Rio Grande do Sul, Brazil	Data collection	
Paulo Victor Lopes	Lopes		MD	Hospital de Clinicas de Porto Alegre	Porto Alegre, Rio Grande do Sul, Brazil	Data collection	
Sara	Torres Y Moreno Batista		MD	Hospital de Clinicas de Porto Alegre	Porto Alegre, Rio Grande do Sul, Brazil	Data collection	
Elveni Teresinha	Agnes Schardong		MD	Hospital de Clinicas de Porto Alegre	Porto Alegre, Rio Grande do Sul, Brazil	Data collection	

Supplemental Online Content: Nonauthor Collaborators
*First name, last name, and suffix (if applicable) are required and will appear in PubMed.

*First Name and Middle Initial(s)	*Last Name	*Suffix (eg, Jr, III)	Academic Degrees	Institution	Location (city, state/province, country)	Role or Contribution, eg, chair, principal investigator	Group (if more than 1 Group listed in the byline) and/or Subgroup (eg, Steering Committee)
Daniel Luiz	Alcântara Nascimento Amorim		MS	Hospital de Clinicas de Porto Alegre	Porto Alegre, Rio Grande do Sul, Brazil	Data collection	
Marina	Almeida Brandão		MD	Hospital de Clinicas de Porto Alegre	Porto Alegre, Rio Grande do Sul, Brazil	Data collection	
Gabriel	Azeredo de Magalhaes		MD	Hospital de Clinicas de Porto Alegre	Porto Alegre, Rio Grande do Sul, Brazil	Data collection	
Emanoel	Baticini Montanari		MD	Hospital de Clinicas de Porto Alegre	Porto Alegre, Rio Grande do Sul, Brazil	Data collection	
Natália	Bender Führ		MD	Hospital de Clinicas de Porto Alegre	Porto Alegre, Rio Grande do Sul, Brazil	Data collection	
Henrique	Bertin Rojas		MD	Hospital de Clinicas de Porto Alegre	Porto Alegre, Rio Grande do Sul, Brazil	Data collection	
Isabela	Bezerra Lumambo		MD	Hospital de Clinicas de Porto Alegre	Porto Alegre, Rio Grande do Sul, Brazil	Data collection	
Crystal	Campos Teixeira		MD	Hospital de Clinicas de Porto Alegre	Porto Alegre, Rio Grande do Sul, Brazil	Data collection	
Alisson	Carvalho de Freitas		MD	Hospital de Clinicas de Porto Alegre	Porto Alegre, Rio Grande do Sul, Brazil	Data collection	
Débora	Castro Ehlert		MD	Hospital de Clinicas de Porto Alegre	Porto Alegre, Rio Grande do Sul, Brazil	Data collection	
João Pedro	Chiqueto de Araújo		MD	Hospital de Clinicas de Porto Alegre	Porto Alegre, Rio Grande do Sul, Brazil	Data collection	
Kellen	Correa		MD	Hospital de Clinicas de Porto Alegre	Porto Alegre, Rio Grande do Sul, Brazil	Data collection	
Clara	Correia de Siracusa		MD	Hospital de Clinicas de Porto Alegre	Porto Alegre, Rio Grande do Sul, Brazil	Data collection	
Agenor Manoel	da Silva		MD	Hospital de Clinicas de Porto Alegre	Porto Alegre, Rio Grande do Sul, Brazil	Data collection	
Gérson Luís	da Silva Nunes		MD	Hospital de Clinicas de Porto Alegre	Porto Alegre, Rio Grande do Sul, Brazil	Data collection	

Supplemental Online Content: Nonauthor Collaborators
*First name, last name, and suffix (if applicable) are required and will appear in PubMed.

*First Name and Middle Initial(s)	*Last Name	*Suffix (eg, Jr, III)	Academic Degrees	Institution	Location (city, state/province, country)	Role or Contribution, eg, chair, principal investigator	Group (if more than 1 Group listed in the byline) and/or Subgroup (eg, Steering Committee)
Karine	Dal Prá		MD	Hospital de Clinicas de Porto Alegre	Porto Alegre, Rio Grande do Sul, Brazil	Data collection	
Renan Israel	De Souza Carvalho		MD	Hospital de Clinicas de Porto Alegre	Porto Alegre, Rio Grande do Sul, Brazil	Data collection	
Luiz Henrique	Dóro Pereira		MD	Hospital de Clinicas de Porto Alegre	Porto Alegre, Rio Grande do Sul, Brazil	Data collection	
Julia	Dullius Oliveira		MD	Hospital de Clinicas de Porto Alegre	Porto Alegre, Rio Grande do Sul, Brazil	Data collection	
Adriana	Fernandes Urgell		MD	Hospital de Clinicas de Porto Alegre	Porto Alegre, Rio Grande do Sul, Brazil	Data collection	
Vera Lucia	Ferreira da Rosa		MD	Hospital de Clinicas de Porto Alegre	Porto Alegre, Rio Grande do Sul, Brazil	Data collection	
Fernanda	Garske Almansa		MD	Hospital de Clinicas de Porto Alegre	Porto Alegre, Rio Grande do Sul, Brazil	Data collection	
Filipe	Gimenes Renck		MD	Hospital de Clinicas de Porto Alegre	Porto Alegre, Rio Grande do Sul, Brazil	Data collection	
Lara	Gonçalves Louzada		MD	Hospital de Clinicas de Porto Alegre	Porto Alegre, Rio Grande do Sul, Brazil	Data collection	
Natascha	Kokay Nepomuceno		MD	Hospital de Clinicas de Porto Alegre	Porto Alegre, Rio Grande do Sul, Brazil	Data collection	
Ana Carolina	Kuwer Bugin		MD	Hospital de Clinicas de Porto Alegre	Porto Alegre, Rio Grande do Sul, Brazil	Data collection	
Ana Paula	Limberger		MD	Hospital de Clinicas de Porto Alegre	Porto Alegre, Rio Grande do Sul, Brazil	Data collection	
Lucas	Lucas Rossetto		MD	Hospital de Clinicas de Porto Alegre	Porto Alegre, Rio Grande do Sul, Brazil	Data collection	
Pedro	Mansur		MD	Hospital de Clinicas de Porto Alegre	Porto Alegre, Rio Grande do Sul, Brazil	Data collection	
Rubens	Matos Maia		MD	Hospital de Clinicas de Porto Alegre	Porto Alegre, Rio Grande do Sul, Brazil	Data collection	

Supplemental Online Content: Nonauthor Collaborators
*First name, last name, and suffix (if applicable) are required and will appear in PubMed.

*First Name and Middle Initial(s)	*Last Name	*Suffix (eg, Jr, III)	Academic Degrees	Institution	Location (city, state/province, country)	Role or Contribution, eg, chair, principal investigator	Group (if more than 1 Group listed in the byline) and/or Subgroup (eg, Steering Committee)
Otavio	Meira Freitas		MD	Hospital de Clinicas de Porto Alegre	Porto Alegre, Rio Grande do Sul, Brazil	Data collection	
Matheus	Niches		MD	Hospital de Clinicas de Porto Alegre	Porto Alegre, Rio Grande do Sul, Brazil	Data collection	
Rafael	Nicolaidis		MD	Hospital de Clinicas de Porto Alegre	Porto Alegre, Rio Grande do Sul, Brazil	Data collection	
Lucas	Odacir Graciolli		MD	Hospital de Clinicas de Porto Alegre	Porto Alegre, Rio Grande do Sul, Brazil	Data collection	
Lucas	Oliveira Junrqueira e Silva		MD	Hospital de Clinicas de Porto Alegre	Porto Alegre, Rio Grande do Sul, Brazil	Data collection	
Mariana	Pessini		MD	Hospital de Clinicas de Porto Alegre	Porto Alegre, Rio Grande do Sul, Brazil	Data collection	
Eduardo	Porto Santos		MS	Hospital de Clinicas de Porto Alegre	Porto Alegre, Rio Grande do Sul, Brazil	Data collection	
Lucas	Quadros Antoniazzi		MS	Hospital de Clinicas de Porto Alegre	Porto Alegre, Rio Grande do Sul, Brazil	Data collection	
Gabriella	Ribeiro Dias		MD	Hospital de Clinicas de Porto Alegre	Porto Alegre, Rio Grande do Sul, Brazil	Data collection	
Natali	Rocha de araujo		MD	Hospital de Clinicas de Porto Alegre	Porto Alegre, Rio Grande do Sul, Brazil	Data collection	
Janilton	Rodrigues Bujes		MD	Hospital de Clinicas de Porto Alegre	Porto Alegre, Rio Grande do Sul, Brazil	Data collection	
Lucas	Rodrigues de Oliveira		MD	Hospital de Clinicas de Porto Alegre	Porto Alegre, Rio Grande do Sul, Brazil	Data collection	
Dieg	Rodrigues dos Santos		MD	Hospital de Clinicas de Porto Alegre	Porto Alegre, Rio Grande do Sul, Brazil	Data collection	
Paulo Renato	Rosales	Jr	MD	Hospital de Clinicas de Porto Alegre	Porto Alegre, Rio Grande do Sul, Brazil	Data collection	
Matheus	Sacco Gomes		MD	Hospital de Clinicas de Porto Alegre	Porto Alegre, Rio Grande do Sul, Brazil	Data collection	

Supplemental Online Content: Nonauthor Collaborators
*First name, last name, and suffix (if applicable) are required and will appear in PubMed.

*First Name and Middle Initial(s)	*Last Name	*Suffix (eg, Jr, III)	Academic Degrees	Institution	Location (city, state/province, country)	Role or Contribution, eg, chair, principal investigator	Group (if more than 1 Group listed in the byline) and/or Subgroup (eg, Steering Committee)
Raoli	Scheidemantel Wagner		MD	Hospital de Clinicas de Porto Alegre	Porto Alegre, Rio Grande do Sul, Brazil	Data collection	
Lucas	Seferin Finardi		MD	Hospital de Clinicas de Porto Alegre	Porto Alegre, Rio Grande do Sul, Brazil	Data collection	
Caroline	Silva Porto Peixoto		MD	Hospital de Clinicas de Porto Alegre	Porto Alegre, Rio Grande do Sul, Brazil	Data collection	
Nathalia	Soares Meier		MD	Hospital de Clinicas de Porto Alegre	Porto Alegre, Rio Grande do Sul, Brazil	Data collection	
Luan	Solei Flores Canteiro		MD	Hospital de Clinicas de Porto Alegre	Porto Alegre, Rio Grande do Sul, Brazil	Data collection	
Vicente	Stolnik Borges		MD	Hospital de Clinicas de Porto Alegre	Porto Alegre, Rio Grande do Sul, Brazil	Data collection	
Alice Eloisa	Szlachta		MD	Hospital de Clinicas de Porto Alegre	Porto Alegre, Rio Grande do Sul, Brazil	Data collection	
Maria Angélica	Vieira Lizama		MD	Hospital de Clinicas de Porto Alegre	Porto Alegre, Rio Grande do Sul, Brazil	Data collection	
Victoria Maria	Coelho Macedo		MS	Hospital de Messejana	Fortaleza, Ceara, Brazil	Data collection	
Luiza	Alencar Moura		MD	Hospital de Messejana	Fortaleza, Ceara, Brazil	Data collection	
Francisco	Alves Lima		MD	Hospital de Messejana	Fortaleza, Ceara, Brazil	Data collection	
Jose	Barreto	Jr	MD	Hospital de Messejana	Fortaleza, Ceara, Brazil	Data collection	
Douglas	Borges Da Costa Filho		MD	Hospital de Messejana	Fortaleza, Ceara, Brazil	Data collection	
Laio	Cardoso De Oliveira		MS	Hospital de Messejana	Fortaleza, Ceara, Brazil	Data collection	
Antonio	Davi		MD	Hospital de Messejana	Fortaleza, Ceara, Brazil	Data collection	
Weverson	De Abreu Lima		MD	Hospital de Messejana	Fortaleza, Ceara, Brazil	Data collection	
Andrezza Maria	de Carvalho Pereira		MS	Hospital de Messejana	Fortaleza, Ceara, Brazil	Data collection	
Felipe	De Menezes Cunha		MD	Hospital de Messejana	Fortaleza, Ceara, Brazil	Data collection	

Supplemental Online Content: Nonauthor Collaborators
*First name, last name, and suffix (if applicable) are required and will appear in PubMed.

*First Name and Middle Initial(s)	*Last Name	*Suffix (eg, Jr, III)	Academic Degrees	Institution	Location (city, state/province, country)	Role or Contribution, eg, chair, principal investigator	Group (if more than 1 Group listed in the byline) and/or Subgroup (eg, Steering Committee)
Lucas	De Vasconcellos Fonteles Teixeira		MD	Hospital de Messejana	Fortaleza, Ceara, Brazil	Data collection	
Gutemberg	Do Nascimento Oliveira		MD	Hospital de Messejana	Fortaleza, Ceara, Brazil	Data collection	
Mattheus	Gondim Muniz		MD	Hospital de Messejana	Fortaleza, Ceara, Brazil	Data collection	
Samer	Heluany Khoury		MD	Hospital de Messejana	Fortaleza, Ceara, Brazil	Data collection	
Kelvio	Lins		MD	Hospital de Messejana	Fortaleza, Ceara, Brazil	Data collection	
Juan	Miguel Cosquillo Valdivia		MD	Hospital de Messejana	Fortaleza, Ceara, Brazil	Data collection	
Lara	Miranda Rodrigues Da Cunha		MD	Hospital de Messejana	Fortaleza, Ceara, Brazil	Data collection	
Emmanuella	Passos Chaves Rocha		MD	Hospital de Messejana	Fortaleza, Ceara, Brazil	Data collection	
Isabelle	Rodrigues Schramm		MD	Hospital de Messejana	Fortaleza, Ceara, Brazil	Data collection	
Bianca	Rohsner Bezerra		MD	Hospital de Messejana	Fortaleza, Ceara, Brazil	Data collection	
Thais	Saraiva Leao Cunha		MD	Hospital de Messejana	Fortaleza, Ceara, Brazil	Data collection	
Angelica	Sauthier		MD	Hospital de Messejana	Fortaleza, Ceara, Brazil	Data collection	
Thiago	Silva Teixeira		MD	Hospital de Messejana	Fortaleza, Ceara, Brazil	Data collection	
Lucas	Soares Coelho Marrocos		MD	Hospital de Messejana	Fortaleza, Ceara, Brazil	Data collection	
Jadson	Soares Laudelino		MD	Hospital de Messejana	Fortaleza, Ceara, Brazil	Data collection	

Supplemental Online Content: Nonauthor Collaborators
*First name, last name, and suffix (if applicable) are required and will appear in PubMed.

***First Name and Middle Initial(s)**	***Last Name**	***Suffix (eg, Jr, III)**	Academic Degrees	Institution	Location (city, state/province, country)	Role or Contribution, eg, chair, principal investigator	Group (if more than 1 Group listed in the byline) and/or Subgroup (eg, Steering Committee)
Leticia	Sucupira Cristino		MD	Hospital de Messejana	Fortaleza, Ceara, Brazil	Data collection	
Clarissa	Thiers		MD	Hospital de Messejana	Fortaleza, Ceara, Brazil	Data collection	
Marina	Almeida Brandão		MD	Hospital de Pronto Socorro	Porto Alegre, Rio Grande do Sul, Brazil	Data collection	
Natália	Bender Fuhr		MD	Hospital de Pronto Socorro	Porto Alegre, Rio Grande do Sul, Brazil	Data collection	
João Vitor	de Camargo Zimmer Vilhena		MD	Hospital de Pronto Socorro	Porto Alegre, Rio Grande do Sul, Brazil	Data collection	
Douglas	Duarte da Rosa		MD	Hospital de Pronto Socorro	Porto Alegre, Rio Grande do Sul, Brazil	Data collection	
Lara	Gonçalves Louzada		MD	Hospital de Pronto Socorro	Porto Alegre, Rio Grande do Sul, Brazil	Data collection	
Gabriel	Müller De Bortoli		MD	Hospital de Pronto Socorro	Porto Alegre, Rio Grande do Sul, Brazil	Data collection	
Mariana	Pessini		MD	Hospital de Pronto Socorro	Porto Alegre, Rio Grande do Sul, Brazil	Data collection	
Luciane	Reis Volkart		MD	Hospital de Pronto Socorro	Porto Alegre, Rio Grande do Sul, Brazil	Data collection	
Gabriella	Ribeiro Dias		MD	Hospital de Pronto Socorro	Porto Alegre, Rio Grande do Sul, Brazil	Data collection	
Leonardo	Sanches Nunes		MD	Hospital de Pronto Socorro	Porto Alegre, Rio Grande do Sul, Brazil	Data collection	
Leonardo	Serena de Morais		MD	Hospital de Pronto Socorro	Porto Alegre, Rio Grande do Sul, Brazil	Data collection	
Gabriel	Seroiska		MS	Hospital de Pronto Socorro	Porto Alegre, Rio Grande do Sul, Brazil	Data collection	
Luan	Solei Flores Canteiro		MD	Hospital de Pronto Socorro	Porto Alegre, Rio Grande do Sul, Brazil	Data collection	

Supplemental Online Content: Nonauthor Collaborators
*First name, last name, and suffix (if applicable) are required and will appear in PubMed.

*First Name and Middle Initial(s)	*Last Name	*Suffix (eg, Jr, III)	Academic Degrees	Institution	Location (city, state/province, country)	Role or Contribution, eg, chair, principal investigator	Group (if more than 1 Group listed in the byline) and/or Subgroup (eg, Steering Committee)
Natália	Tomazelli		MS	Hospital de Pronto Socorro	Porto Alegre, Rio Grande do Sul, Brazil	Data collection	
Ana Julia Araujo de Carvalho	Araujo de Carvalho		MD	Hospital de Pronto Socorro	Porto Alegre, Rio Grande do Sul, Brazil	Data collection	
Sofia	Augustin Rota		MS	Hospital de Pronto Socorro	Porto Alegre, Rio Grande do Sul, Brazil	Data collection	
Heloisa	Beckhauser		MD	Hospital de Pronto Socorro	Porto Alegre, Rio Grande do Sul, Brazil	Data collection	
Débora	Castro Ehlert		MD	Hospital de Pronto Socorro	Porto Alegre, Rio Grande do Sul, Brazil	Data collection	
Joao Pedro	Chiqueto de Araujo		MD	Hospital de Pronto Socorro	Porto Alegre, Rio Grande do Sul, Brazil	Data collection	
Natali	da Rocha de Araujo		MD	Hospital de Pronto Socorro	Porto Alegre, Rio Grande do Sul, Brazil	Data collection	
Pedro Henrique	de almeida frencisco		MD	Hospital de Pronto Socorro	Porto Alegre, Rio Grande do Sul, Brazil	Data collection	
Victoria	de Jorge		MS	Hospital de Pronto Socorro	Porto Alegre, Rio Grande do Sul, Brazil	Data collection	
Pedro Alves	do Amaral Mansur		MD	Hospital de Pronto Socorro	Porto Alegre, Rio Grande do Sul, Brazil	Data collection	
Letícia	Emos de Araújo		MD	Hospital de Pronto Socorro	Porto Alegre, Rio Grande do Sul, Brazil	Data collection	
Paulo Victor	Lopes		MD	Hospital de Pronto Socorro	Porto Alegre, Rio Grande do Sul, Brazil	Data collection	
Rubens	Matos Maia		MD	Hospital de Pronto Socorro	Porto Alegre, Rio Grande do Sul, Brazil	Data collection	
Otavio	Meira Freitas		MD	Hospital de Pronto Socorro	Porto Alegre, Rio Grande do Sul, Brazil	Data collection	
Amanda Paim Noya	Paim Noya		MS	Hospital de Pronto Socorro	Porto Alegre, Rio Grande do Sul, Brazil	Data collection	

Supplemental Online Content: Nonauthor Collaborators
*First name, last name, and suffix (if applicable) are required and will appear in PubMed.

*First Name and Middle Initial(s)	*Last Name	*Suffix (eg, Jr, III)	Academic Degrees	Institution	Location (city, state/province, country)	Role or Contribution, eg, chair, principal investigator	Group (if more than 1 Group listed in the byline) and/or Subgroup (eg, Steering Committee)
Paulo Ricardo	Pereira Engrazia		MD	Hospital de Pronto Socorro	Porto Alegre, Rio Grande do Sul, Brazil	Data collection	
Samara	Quoos da Rosa		MS	Hospital de Pronto Socorro	Porto Alegre, Rio Grande do Sul, Brazil	Data collection	
Lucas	Rossetto		MD	Hospital de Pronto Socorro	Porto Alegre, Rio Grande do Sul, Brazil	Data collection	
Adara Saito Goes	Saito Goes		MD	Hospital de Pronto Socorro	Porto Alegre, Rio Grande do Sul, Brazil	Data collection	
Raoli	Scheidemantel Wagner		MD	Hospital de Pronto Socorro	Porto Alegre, Rio Grande do Sul, Brazil	Data collection	
Larissa	Stuermer		MS	Hospital de Pronto Socorro	Porto Alegre, Rio Grande do Sul, Brazil	Data collection	
Vanderson	Tobias Lazaroto		MS	Hospital de Pronto Socorro	Porto Alegre, Rio Grande do Sul, Brazil	Data collection	
Vitor	Amorim de Andrade Câmara		MD	Hospital Estadual de Botucatu UNESP	Sao Paulo, Sao Paulo, Brazil	Data collection	
Thomás	de Souza Patto Marcondes		MD	Hospital Estadual de Botucatu UNESP	Sao Paulo, Sao Paulo, Brazil	Data collection	
Címtia Mitsue Pereira Suzuki	Mitsue Pereira Suzuki		MD	Hospital Estadual de Botucatu UNESP	Sao Paulo, Sao Paulo, Brazil	Data collection	
Igor	Antunes Campinas		MD	Hospital Estadual de Botucatu UNESP	Sao Paulo, Sao Paulo, Brazil	Data collection	
Eduarda	Baccarin Ferrari		MD	Hospital Estadual de Botucatu UNESP	Sao Paulo, Sao Paulo, Brazil	Data collection	
Ana Luíza	Bilória de Aguiar		MD	Hospital Estadual de Botucatu UNESP	Sao Paulo, Sao Paulo, Brazil	Data collection	
Mayara	Bosquê Marangon		MD	Hospital Estadual de Botucatu UNESP	Sao Paulo, Sao Paulo, Brazil	Data collection	
Livia	Cafundo Almeida		MD	Hospital Estadual de Botucatu UNESP	Sao Paulo, Sao Paulo, Brazil	Data collection	

Supplemental Online Content: Nonauthor Collaborators
*First name, last name, and suffix (if applicable) are required and will appear in PubMed.

*First Name and Middle Initial(s)	*Last Name	*Suffix (eg, Jr, III)	Academic Degrees	Institution	Location (city, state/province, country)	Role or Contribution, eg, chair, principal investigator	Group (if more than 1 Group listed in the byline) and/or Subgroup (eg, Steering Committee)
Daniel	Corrêa		MD	Hospital Estadual de Botucatu UNESP	Sao Paulo, Sao Paulo, Brazil	Data collection	
Thiago	Dias Baumgratz		MD	Hospital Estadual de Botucatu UNESP	Sao Paulo, Sao Paulo, Brazil	Data collection	
Matheus	Esteves Pelicer		MD	Hospital Estadual de Botucatu UNESP	Sao Paulo, Sao Paulo, Brazil	Data collection	
Murillo	Favaro		MD	Hospital Estadual de Botucatu UNESP	Sao Paulo, Sao Paulo, Brazil	Data collection	
Edson Luiz	Favero Junior		MD	Hospital Estadual de Botucatu UNESP	Sao Paulo, Sao Paulo, Brazil	Data collection	
Pedro	Fogaça Mateus		MD	Hospital Estadual de Botucatu UNESP	Sao Paulo, Sao Paulo, Brazil	Data collection	
Maria	Guerra		MD	Hospital Estadual de Botucatu UNESP	Sao Paulo, Sao Paulo, Brazil	Data collection	
Natanye	Lemes Matchil		MD	Hospital Estadual de Botucatu UNESP	Sao Paulo, Sao Paulo, Brazil	Data collection	
Marcio Antônio	Marques		MD	Hospital Estadual de Botucatu UNESP	Sao Paulo, Sao Paulo, Brazil	Data collection	
Rodrigo	Martins Carvalho		MD	Hospital Estadual de Botucatu UNESP	Sao Paulo, Sao Paulo, Brazil	Data collection	
Ana Clara	Muraro Bonini		MD	Hospital Estadual de Botucatu UNESP	Sao Paulo, Sao Paulo, Brazil	Data collection	
Manuela	Nazi		MD	Hospital Estadual de Botucatu UNESP	Sao Paulo, Sao Paulo, Brazil	Data collection	
André Marcos	Oliveira		MD	Hospital Estadual de Botucatu UNESP	Sao Paulo, Sao Paulo, Brazil	Data collection	
Letícia	Pedreira de Menezes		MD	Hospital Estadual de Botucatu UNESP	Sao Paulo, Sao Paulo, Brazil	Data collection	
Giovanni	Pedroni		MD	Hospital Estadual de Botucatu UNESP	Sao Paulo, Sao Paulo, Brazil	Data collection	

Supplemental Online Content: Nonauthor Collaborators
*First name, last name, and suffix (if applicable) are required and will appear in PubMed.

***First Name and Middle Initial(s)**	***Last Name**	***Suffix (eg, Jr, III)**	Academic Degrees	Institution	Location (city, state/province, country)	Role or Contribution, eg, chair, principal investigator	Group (if more than 1 Group listed in the byline) and/or Subgroup (eg, Steering Committee)
Ana Flavia	Ribeiro Francisco		MD	Hospital Estadual de Botucatu UNESP	Sao Paulo, Sao Paulo, Brazil	Data collection	
Pedro Ricardo	Rosseti		MD	Hospital Estadual de Botucatu UNESP	Sao Paulo, Sao Paulo, Brazil	Data collection	
Willian	Sacco Altran		MD	Hospital Estadual de Botucatu UNESP	Sao Paulo, Sao Paulo, Brazil	Data collection	
Felipe	Sanches Ferrari		MD	Hospital Estadual de Botucatu UNESP	Sao Paulo, Sao Paulo, Brazil	Data collection	
Sean Hideo	Shirata Lancas		MD	Hospital Estadual de Botucatu UNESP	Sao Paulo, Sao Paulo, Brazil	Data collection	
Michel	Toledo de Souza		MD	Hospital Estadual de Botucatu UNESP	Sao Paulo, Sao Paulo, Brazil	Data collection	
Ingrid Maria	Vieira de Lyra		MD	Hospital Estadual de Botucatu UNESP	Sao Paulo, Sao Paulo, Brazil	Data collection	
Felipe Antonio	Rischini		MD	Hospital Estadual de Botucatu UNESP	Sao Paulo, Sao Paulo, Brazil	Regional Case Manager	
Livia	Andrade Gurgel		MD	Hospital Geral de Fortaleza	Fortaleza, Ceara, Brazil	Data collection	
Mateus	Lopes Moreira		MD	Hospital Geral de Fortaleza	Fortaleza, Ceara, Brazil	Data collection	
Louise	Tahim de Sousa Brasil Othon Sidou		MD	Hospital Geral de Fortaleza	Fortaleza, Ceara, Brazil	Data collection	
Leticia	Aguiar Fonseca		MD	Hospital Geral de Fortaleza	Fortaleza, Ceara, Brazil	Data collection	
Marissa	Barbara Eduardo Souza		MD	Hospital Geral de Fortaleza	Fortaleza, Ceara, Brazil	Data collection	
Renato	Barbosa dos Santos		MD	Hospital Geral de Fortaleza	Fortaleza, Ceara, Brazil	Data collection	
Beatriz	Cabral		MD	Hospital Geral de Fortaleza	Fortaleza, Ceara, Brazil	Data collection	
Joao Vitor	Candido Pimental		MD	Hospital Geral de Fortaleza	Fortaleza, Ceara, Brazil	Data collection	

Supplemental Online Content: Nonauthor Collaborators
*First name, last name, and suffix (if applicable) are required and will appear in PubMed.

*First Name and Middle Initial(s)	*Last Name	*Suffix (eg, Jr, III)	Academic Degrees	Institution	Location (city, state/province, country)	Role or Contribution, eg, chair, principal investigator	Group (if more than 1 Group listed in the byline) and/or Subgroup (eg, Steering Committee)
Luiza	Carla de Medeiros		MD	Hospital Geral de Fortaleza	Fortaleza, Ceara, Brazil	Data collection	
Matheus	Carvalho Vasconcelos		MD	Hospital Geral de Fortaleza	Fortaleza, Ceara, Brazil	Data collection	
Natalia	Chaves Marques		MD	Hospital Geral de Fortaleza	Fortaleza, Ceara, Brazil	Data collection	
Raimunda	da Silva Marcos		MD	Hospital Geral de Fortaleza	Fortaleza, Ceara, Brazil	Data collection	
Victor Hugo	De Alemeida Oliveira		MD	Hospital Geral de Fortaleza	Fortaleza, Ceara, Brazil	Data collection	
Luis	Dias Freires		MD	Hospital Geral de Fortaleza	Fortaleza, Ceara, Brazil	Data collection	
Jade	dos Santos Pires de Carvalho		MD	Hospital Geral de Fortaleza	Fortaleza, Ceara, Brazil	Data collection	
Maria Karoline	Ferreira Santana		MD	Hospital Geral de Fortaleza	Fortaleza, Ceara, Brazil	Data collection	
Giovana	Fischer Neto		MD	Hospital Geral de Fortaleza	Fortaleza, Ceara, Brazil	Data collection	
Talita	Guimaraes Andrade		MD	Hospital Geral de Fortaleza	Fortaleza, Ceara, Brazil	Data collection	
Lilian	Macambira Pinto		MD	Hospital Geral de Fortaleza		Data collection	
Thiago	Maciel Valente		MD	Hospital Geral de Fortaleza	Fortaleza, Ceara, Brazil	Data collection	
Gustavo	Mesquita		MD	Hospital Geral de Fortaleza	Fortaleza, Ceara, Brazil	Data collection	
Isabele	Neves		MD	Hospital Geral de Fortaleza	Fortaleza, Ceara, Brazil	Data collection	
Fernando	Octavio Machado Juca	Neto	MD	Hospital Geral de Fortaleza	Fortaleza, Ceara, Brazil	Data collection	
Marcos	Oliveira		MD	Hospital Geral de Fortaleza	Fortaleza, Ceara, Brazil	Data collection	
Renan	Paiva		MD	Hospital Geral de Fortaleza	Fortaleza, Ceara, Brazil	Data collection	
Joana	Parente		MD	Hospital Geral de Fortaleza	Fortaleza, Ceara, Brazil	Data collection	
Gabriel	Petermann		MD	Hospital Geral de Fortaleza	Fortaleza, Ceara, Brazil	Data collection	
Aline	Ramos		MD	Hospital Geral de Fortaleza	Fortaleza, Ceara, Brazil	Data collection	

Supplemental Online Content: Nonauthor Collaborators
*First name, last name, and suffix (if applicable) are required and will appear in PubMed.

*First Name and Middle Initial(s)	*Last Name	*Suffix (eg, Jr, III)	Academic Degrees	Institution	Location (city, state/province, country)	Role or Contribution, eg, chair, principal investigator	Group (if more than 1 Group listed in the byline) and/or Subgroup (eg, Steering Committee)
Celina	Raquel Moura Rocha		MD	Hospital Geral de Fortaleza	Fortaleza, Ceara, Brazil	Data collection	
Maria Rilda	Rilda Loiola De Vasconcelos		MD	Hospital Geral de Fortaleza	Fortaleza, Ceara, Brazil	Data collection	
Maria das Dores	Rodrigues		MD	Hospital Geral de Fortaleza	Fortaleza, Ceara, Brazil	Data collection	
Maria Hidilma	Rodrigues Muniz		MD	Hospital Geral de Fortaleza	Fortaleza, Ceara, Brazil	Data collection	
Alyne	Silva Araujo		MD	Hospital Geral de Fortaleza	Fortaleza, Ceara, Brazil	Data collection	
Samuel	Silva de Oliveira		MD	Hospital Geral de Fortaleza	Fortaleza, Ceara, Brazil	Data collection	
Isabelle	Teixeira		MD	Hospital Geral de Fortaleza	Fortaleza, Ceara, Brazil	Data collection	
Caio	Ulianov de Lisboa Santos		MD	Hospital Geral de Fortaleza	Fortaleza, Ceara, Brazil	Data collection	
Lucas	Avila de Souza		MD	Hospital Metropolitano Odilon Behrens	Belo Horizonte, Minas Gerais, Brazil	Data collection	
Mariana	Lopes Cancado Lira		MD	Hospital Metropolitano Odilon Behrens	Belo Horizonte, Minas Gerais, Brazil	Data collection	
Matheus	Rodrigues de Oliveira		MD	Hospital Metropolitano Odilon Behrens	Belo Horizonte, Minas Gerais, Brazil	Data collection	
Rafael	Ageu Franklinde Freitas		MD	Hospital Metropolitano Odilon Behrens	Belo Horizonte, Minas Gerais, Brazil	Data collection	
Maria Mariana	Aguiar		MD	Hospital Metropolitano Odilon Behrens	Belo Horizonte, Minas Gerais, Brazil	Data collection	
Mariana	Alcantara Nascimento		MD	Hospital Metropolitano Odilon Behrens	Belo Horizonte, Minas Gerais, Brazil	Data collection	
Diego	Alcantara Santos		MD	Hospital Metropolitano Odilon Behrens	Belo Horizonte, Minas Gerais, Brazil	Data collection	
Thais	Almeida		MD	Hospital Metropolitano Odilon Behrens	Belo Horizonte, Minas Gerais, Brazil	Data collection	

Supplemental Online Content: Nonauthor Collaborators
*First name, last name, and suffix (if applicable) are required and will appear in PubMed.

*First Name and Middle Initial(s)	*Last Name	*Suffix (eg, Jr, III)	Academic Degrees	Institution	Location (city, state/province, country)	Role or Contribution, eg, chair, principal investigator	Group (if more than 1 Group listed in the byline) and/or Subgroup (eg, Steering Committee)
Ana Claudia	Almeida Coelho		MD	Hospital Metropolitano Odilon Behrens	Belo Horizonte, Minas Gerais, Brazil	Data collection	
Jeane	Almeida Prates		MD	Hospital Metropolitano Odilon Behrens	Belo Horizonte, Minas Gerais, Brazil	Data collection	
Flaviano	Alves da Rocha		MD	Hospital Metropolitano Odilon Behrens	Belo Horizonte, Minas Gerais, Brazil	Data collection	
Maria Julia	Alves de Souza		MD	Hospital Metropolitano Odilon Behrens	Belo Horizonte, Minas Gerais, Brazil	Data collection	
Danielle	Alves Vieira		MD	Hospital Metropolitano Odilon Behrens	Belo Horizonte, Minas Gerais, Brazil	Data collection	
Larissa	Amora Gomes		MD	Hospital Metropolitano Odilon Behrens	Belo Horizonte, Minas Gerais, Brazil	Data collection	
Marianna	Amorim Antunes		MD	Hospital Metropolitano Odilon Behrens	Belo Horizonte, Minas Gerais, Brazil	Data collection	
Anna Luisa	Amorim Chaves		MD	Hospital Metropolitano Odilon Behrens	Belo Horizonte, Minas Gerais, Brazil	Data collection	
Gessica	Antonia Fernandes		MD	Hospital Metropolitano Odilon Behrens	Belo Horizonte, Minas Gerais, Brazil	Data collection	
Caritas	Antunes Lacerda		MD	Hospital Metropolitano Odilon Behrens	Belo Horizonte, Minas Gerais, Brazil	Data collection	
Lucas	Araujo Carineiro de Abreu		MD	Hospital Metropolitano Odilon Behrens	Belo Horizonte, Minas Gerais, Brazil	Data collection	
Pedro Henrique	Arcanjo Alvarenga		Medical Student	Hospital Metropolitano Odilon Behrens	Belo Horizonte, Minas Gerais, Brazil	Data collection	
Larissa	Assis Abreu		MD	Hospital Metropolitano Odilon Behrens	Belo Horizonte, Minas Gerais, Brazil	Data collection	
Jose Miguel	Assis Borges		MD	Hospital Metropolitano Odilon Behrens	Belo Horizonte, Minas Gerais, Brazil	Data collection	
Jose Miguel	Assis Borges		MD	Hospital Metropolitano Odilon Behrens	Belo Horizonte, Minas Gerais, Brazil	Data collection	

Supplemental Online Content: Nonauthor Collaborators
*First name, last name, and suffix (if applicable) are required and will appear in PubMed.

*First Name and Middle Initial(s)	*Last Name	*Suffix (eg, Jr, III)	Academic Degrees	Institution	Location (city, state/province, country)	Role or Contribution, eg, chair, principal investigator	Group (if more than 1 Group listed in the byline) and/or Subgroup (eg, Steering Committee)
Guilherme	Avila Abrahao Reis		MD	Hospital Metropolitano Odilon Behrens	Belo Horizonte, Minas Gerais, Brazil	Data collection	
Ana Carolina	Barreto Martyn Costa		MD	Hospital Metropolitano Odilon Behrens	Belo Horizonte, Minas Gerais, Brazil	Data collection	
Hugo	Barros Alves		MD	Hospital Metropolitano Odilon Behrens	Belo Horizonte, Minas Gerais, Brazil	Data collection	
Paulo Henrique	Barros Valente		MD	Hospital Metropolitano Odilon Behrens	Belo Horizonte, Minas Gerais, Brazil	Data collection	
Julia	Barroso Chiari		MD	Hospital Metropolitano Odilon Behrens	Belo Horizonte, Minas Gerais, Brazil	Data collection	
Renata	Bastos Peres		Nurse	Hospital Metropolitano Odilon Behrens	Belo Horizonte, Minas Gerais, Brazil	Data collection	
Flavio Henrique	Bernardes Papa		MD	Hospital Metropolitano Odilon Behrens	Belo Horizonte, Minas Gerais, Brazil	Data collection	
Tatiana Roberta	Bogutchi Sarubi		MD	Hospital Metropolitano Odilon Behrens	Belo Horizonte, Minas Gerais, Brazil	Data collection	
Silvia	Bouissou Morais Soares		MD	Hospital Metropolitano Odilon Behrens	Belo Horizonte, Minas Gerais, Brazil	Data collection	
Hanrafel Geraldo	Caetano da Silva		MD	Hospital Metropolitano Odilon Behrens	Belo Horizonte, Minas Gerais, Brazil	Data collection	
Luisa	Campos Martins		MD	Hospital Metropolitano Odilon Behrens	Belo Horizonte, Minas Gerais, Brazil	Data collection	
Luisa	Campos Martins		MD	Hospital Metropolitano Odilon Behrens	Belo Horizonte, Minas Gerais, Brazil	Data collection	
Jose Sergio	Carriero Junior		MD	Hospital Metropolitano Odilon Behrens	Belo Horizonte, Minas Gerais, Brazil	Data collection	
Maria Eliene	Carvalho Barreiros		MD	Hospital Metropolitano Odilon Behrens	Belo Horizonte, Minas Gerais, Brazil	Data collection	
Fabio	Carvalho Fonseca		MD	Hospital Metropolitano Odilon Behrens	Belo Horizonte, Minas Gerais, Brazil	Data collection	

Supplemental Online Content: Nonauthor Collaborators
*First name, last name, and suffix (if applicable) are required and will appear in PubMed.

*First Name and Middle Initial(s)	*Last Name	*Suffix (eg, Jr, III)	Academic Degrees	Institution	Location (city, state/province, country)	Role or Contribution, eg, chair, principal investigator	Group (if more than 1 Group listed in the byline) and/or Subgroup (eg, Steering Committee)
Marcos Henrique	Catizani Primola Faria		MD	Hospital Metropolitano Odilon Behrens	Belo Horizonte, Minas Gerais, Brazil	Data collection	
Jose Reinaldo	Correa Roveda		MD	Hospital Metropolitano Odilon Behrens	Belo Horizonte, Minas Gerais, Brazil	Data collection	
Jose Reinaldo	Correa Roveda		MD	Hospital Metropolitano Odilon Behrens	Belo Horizonte, Minas Gerais, Brazil	Data collection	
Raphaela	Costa Sales		MD	Hospital Metropolitano Odilon Behrens	Belo Horizonte, Minas Gerais, Brazil	Data collection	
Gustavo	Couto Pereira da Silva		MD	Hospital Metropolitano Odilon Behrens	Belo Horizonte, Minas Gerais, Brazil	Data collection	
Jose Alexandre	Cruz Neto		MD	Hospital Metropolitano Odilon Behrens	Belo Horizonte, Minas Gerais, Brazil	Data collection	
Patricia Vivianne	da Silva Pedra		MD	Hospital Metropolitano Odilon Behrens	Belo Horizonte, Minas Gerais, Brazil	Data collection	
Karen	de Almeida Prado Rabello		MD	Hospital Metropolitano Odilon Behrens	Belo Horizonte, Minas Gerais, Brazil	Data collection	
Erika	de Almeida Santos Quadros		MD	Hospital Metropolitano Odilon Behrens	Belo Horizonte, Minas Gerais, Brazil	Data collection	
Thamyres	de Carvalho Rufato		MD	Hospital Metropolitano Odilon Behrens	Belo Horizonte, Minas Gerais, Brazil	Data collection	
Hannah	de Castro almeida		MD	Hospital Metropolitano Odilon Behrens	Belo Horizonte, Minas Gerais, Brazil	Data collection	
Ana Clara	de Castro e Braga		MD	Hospital Metropolitano Odilon Behrens	Belo Horizonte, Minas Gerais, Brazil	Data collection	
Eva Maria	DE FATIMA SORRENTINO DE PAULA		MD	Hospital Metropolitano Odilon Behrens	Belo Horizonte, Minas Gerais, Brazil	Data collection	
Daniel	de Lima Ruas		MD	Hospital Metropolitano Odilon Behrens	Belo Horizonte, Minas Gerais, Brazil	Data collection	

Supplemental Online Content: Nonauthor Collaborators
*First name, last name, and suffix (if applicable) are required and will appear in PubMed.

*First Name and Middle Initial(s)	*Last Name	*Suffix (eg, Jr, III)	Academic Degrees	Institution	Location (city, state/province, country)	Role or Contribution, eg, chair, principal investigator	Group (if more than 1 Group listed in the byline) and/or Subgroup (eg, Steering Committee)
Yulle	de Oliveira Martins		MD	Hospital Metropolitano Odilon Behrens	Belo Horizonte, Minas Gerais, Brazil	Data collection	
Marina	de Oliveira Rabello		MD	Hospital Metropolitano Odilon Behrens	Belo Horizonte, Minas Gerais, Brazil	Data collection	
Joao Roberto	de Souza		MD	Hospital Metropolitano Odilon Behrens	Belo Horizonte, Minas Gerais, Brazil	Data collection	
Crisellen	Delogo Sinete		MD	Hospital Metropolitano Odilon Behrens	Belo Horizonte, Minas Gerais, Brazil	Data collection	
Lucas Ismael	Dias Pereira		MD	Hospital Metropolitano Odilon Behrens	Belo Horizonte, Minas Gerais, Brazil	Data collection	
Luisa	Diniz Reis		MD	Hospital Metropolitano Odilon Behrens	Belo Horizonte, Minas Gerais, Brazil	Data collection	
Lucas	Dornelas Moreira de Melo		MD	Hospital Metropolitano Odilon Behrens	Belo Horizonte, Minas Gerais, Brazil	Data collection	
Julia	Duarte Ruiz Costa		MD	Hospital Metropolitano Odilon Behrens	Belo Horizonte, Minas Gerais, Brazil	Data collection	
Naiara Patricia	Fagundes Bonardi		MD	Hospital Metropolitano Odilon Behrens	Belo Horizonte, Minas Gerais, Brazil	Data collection	
Mariana	Felice Machado		MD	Hospital Metropolitano Odilon Behrens	Belo Horizonte, Minas Gerais, Brazil	Data collection	
Pedro Ferrari	Ferrari Sales da Cunha		MD	Hospital Metropolitano Odilon Behrens	Belo Horizonte, Minas Gerais, Brazil	Data collection	
Cirilo Jose	Ferreira	Neto	MD	Hospital Metropolitano Odilon Behrens	Belo Horizonte, Minas Gerais, Brazil	Data collection	
Mayara	Ferreira dos Reis		MD	Hospital Metropolitano Odilon Behrens	Belo Horizonte, Minas Gerais, Brazil	Data collection	
Vitor Augusto	Ferreira Herculano		MD	Hospital Metropolitano Odilon Behrens	Belo Horizonte, Minas Gerais, Brazil	Data collection	
Sara	Fia Giori		MD	Hospital Metropolitano Odilon Behrens	Belo Horizonte, Minas Gerais, Brazil	Data collection	

Supplemental Online Content: Nonauthor Collaborators
*First name, last name, and suffix (if applicable) are required and will appear in PubMed.

*First Name and Middle Initial(s)	*Last Name	*Suffix (eg, Jr, III)	Academic Degrees	Institution	Location (city, state/province, country)	Role or Contribution, eg, chair, principal investigator	Group (if more than 1 Group listed in the byline) and/or Subgroup (eg, Steering Committee)
Millena	Figueiredo Miranda		MD	Hospital Metropolitano Odilon Behrens	Belo Horizonte, Minas Gerais, Brazil	Data collection	
Breno	Filippo Rezende		MD	Hospital Metropolitano Odilon Behrens	Belo Horizonte, Minas Gerais, Brazil	Data collection	
Pedro Gabriel	Fonseca Silva Rocha		MD	Hospital Metropolitano Odilon Behrens	Belo Horizonte, Minas Gerais, Brazil	Data collection	
Julia	Fontes Parizzi		MD	Hospital Metropolitano Odilon Behrens	Belo Horizonte, Minas Gerais, Brazil	Data collection	
Milena	Freire Abu Kamel		MD	Hospital Metropolitano Odilon Behrens	Belo Horizonte, Minas Gerais, Brazil	Data collection	
Carolina	Freitas Campos		PT	Hospital Metropolitano Odilon Behrens	Belo Horizonte, Minas Gerais, Brazil	Data collection	
Sarah	Freitas Ferreira		MD	Hospital Metropolitano Odilon Behrens	Belo Horizonte, Minas Gerais, Brazil	Data collection	
Ana Paula	Furtado Santos		MD	Hospital Metropolitano Odilon Behrens	Belo Horizonte, Minas Gerais, Brazil	Data collection	
Ursula	Gama Pimenta Murta		MD	Hospital Metropolitano Odilon Behrens	Belo Horizonte, Minas Gerais, Brazil	Data collection	
Angela	Gil Patrus Pena		MD	Hospital Metropolitano Odilon Behrens	Belo Horizonte, Minas Gerais, Brazil	Data collection	
Amanda	Goncalves Cerqueira		MD	Hospital Metropolitano Odilon Behrens	Belo Horizonte, Minas Gerais, Brazil	Data collection	
Sofia	Gonzaga Garcia		MD	Hospital Metropolitano Odilon Behrens	Belo Horizonte, Minas Gerais, Brazil	Data collection	
Luiza	Gouvea Dutra Teixeira		MD	Hospital Metropolitano Odilon Behrens	Belo Horizonte, Minas Gerais, Brazil	Data collection	
Celso	Groppo de Oliveira	Jr	MD	Hospital Metropolitano Odilon Behrens	Belo Horizonte, Minas Gerais, Brazil	Data collection	
Samuel	Guimaraes Vitarelli		MD	Hospital Metropolitano Odilon Behrens	Belo Horizonte, Minas Gerais, Brazil	Data collection	

Supplemental Online Content: Nonauthor Collaborators
*First name, last name, and suffix (if applicable) are required and will appear in PubMed.

*First Name and Middle Initial(s)	*Last Name	*Suffix (eg, Jr, III)	Academic Degrees	Institution	Location (city, state/province, country)	Role or Contribution, eg, chair, principal investigator	Group (if more than 1 Group listed in the byline) and/or Subgroup (eg, Steering Committee)
Pedro	Henrique de Oliveira Queiroz		MD	Hospital Metropolitano Odilon Behrens	Belo Horizonte, Minas Gerais, Brazil	Data collection	
Renata	Holanda Muniz Falcao Soares		MD	Hospital Metropolitano Odilon Behrens	Belo Horizonte, Minas Gerais, Brazil	Data collection	
Paola	Isabelle Mariano		MD	Hospital Metropolitano Odilon Behrens	Belo Horizonte, Minas Gerais, Brazil	Data collection	
Jose Expedito	Jannotti Neto		MD	Hospital Metropolitano Odilon Behrens	Belo Horizonte, Minas Gerais, Brazil	Data collection	
Mariana	Lacerda Silva		MD	Hospital Metropolitano Odilon Behrens	Belo Horizonte, Minas Gerais, Brazil	Data collection	
Carolina	Laignier		MD	Hospital Metropolitano Odilon Behrens	Belo Horizonte, Minas Gerais, Brazil	Data collection	
Elier	Lamas Teixeira		MD	Hospital Metropolitano Odilon Behrens	Belo Horizonte, Minas Gerais, Brazil	Data collection	
Izabela	Lara Borem		MD	Hospital Metropolitano Odilon Behrens	Belo Horizonte, Minas Gerais, Brazil	Data collection	
Gabriel	Leao Froes		MD	Hospital Metropolitano Odilon Behrens	Belo Horizonte, Minas Gerais, Brazil	Data collection	
Ana Claudia	Leite da Silva Ferreira		MD	Hospital Metropolitano Odilon Behrens	Belo Horizonte, Minas Gerais, Brazil	Data collection	
Bernardo	Lentz da Silveira Monteiro		MD	Hospital Metropolitano Odilon Behrens	Belo Horizonte, Minas Gerais, Brazil	Data collection	
Ana Carolina	Lobato Saldanha		MD	Hospital Metropolitano Odilon Behrens	Belo Horizonte, Minas Gerais, Brazil	Data collection	
Isabela	Lopes Costa		MD	Hospital Metropolitano Odilon Behrens	Belo Horizonte, Minas Gerais, Brazil	Data collection	
Fernanda	Loureiro Ignacio		MD	Hospital Metropolitano Odilon Behrens	Belo Horizonte, Minas Gerais, Brazil	Data collection	

Supplemental Online Content: Nonauthor Collaborators
*First name, last name, and suffix (if applicable) are required and will appear in PubMed.

*First Name and Middle Initial(s)	*Last Name	*Suffix (eg, Jr, III)	Academic Degrees	Institution	Location (city, state/province, country)	Role or Contribution, eg, chair, principal investigator	Group (if more than 1 Group listed in the byline) and/or Subgroup (eg, Steering Committee)
Sarah	Maciel Silva		MD	Hospital Metropolitano Odilon Behrens	Belo Horizonte, Minas Gerais, Brazil	Data collection	
Daniela	Magalhaes Moreira dos Santos		MD	Hospital Metropolitano Odilon Behrens	Belo Horizonte, Minas Gerais, Brazil	Data collection	
Sara	Magro Borigato		MD	Hospital Metropolitano Odilon Behrens	Belo Horizonte, Minas Gerais, Brazil	Data collection	
Izabella	Marcal Macedo		MD	Hospital Metropolitano Odilon Behrens	Belo Horizonte, Minas Gerais, Brazil	Data collection	
Joao Marcos	Marcos Coelho de Azevedo		MD	Hospital Metropolitano Odilon Behrens	Belo Horizonte, Minas Gerais, Brazil	Data collection	
Paola	Mariano		MD	Hospital Metropolitano Odilon Behrens	Belo Horizonte, Minas Gerais, Brazil	Data collection	
Warlei	Martins		MD	Hospital Metropolitano Odilon Behrens	Belo Horizonte, Minas Gerais, Brazil	Data collection	
Lara Maria	Martins Lima		MD	Hospital Metropolitano Odilon Behrens	Belo Horizonte, Minas Gerais, Brazil	Data collection	
Camila	Martins Lopes		MD	Hospital Metropolitano Odilon Behrens	Belo Horizonte, Minas Gerais, Brazil	Data collection	
Iara	Martins Lopes		MD	Hospital Metropolitano Odilon Behrens	Belo Horizonte, Minas Gerais, Brazil	Data collection	
Neuza	Martins Stopa da Costa		MD	Hospital Metropolitano Odilon Behrens	Belo Horizonte, Minas Gerais, Brazil	Data collection	
Oliveira	Martins Terra		MD	Hospital Metropolitano Odilon Behrens	Belo Horizonte, Minas Gerais, Brazil	Data collection	
Alessandra	Marzano		MD	Hospital Metropolitano Odilon Behrens	Belo Horizonte, Minas Gerais, Brazil	Data collection	
Caroline Luisa	Massote Couto		MD	Hospital Metropolitano Odilon Behrens	Belo Horizonte, Minas Gerais, Brazil	Data collection	
Larissa	Matos Ventura		MD	Hospital Metropolitano Odilon Behrens	Belo Horizonte, Minas Gerais, Brazil	Data collection	

Supplemental Online Content: Nonauthor Collaborators
*First name, last name, and suffix (if applicable) are required and will appear in PubMed.

*First Name and Middle Initial(s)	*Last Name	*Suffix (eg, Jr, III)	Academic Degrees	Institution	Location (city, state/province, country)	Role or Contribution, eg, chair, principal investigator	Group (if more than 1 Group listed in the byline) and/or Subgroup (eg, Steering Committee)
Rodrigo	Medrado Pereira Lopes		MD	Hospital Metropolitano Odilon Behrens	Belo Horizonte, Minas Gerais, Brazil	Data collection	
Eduardo Henrique	Meneses Prado		MD	Hospital Metropolitano Odilon Behrens	Belo Horizonte, Minas Gerais, Brazil	Data collection	
Leticia	Meyer Ulhoa		MD	Hospital Metropolitano Odilon Behrens	Belo Horizonte, Minas Gerais, Brazil	Data collection	
Joaquim Victor	Miranda Dias		MD	Hospital Metropolitano Odilon Behrens	Belo Horizonte, Minas Gerais, Brazil	Data collection	
Lara	Miranda Rodrigues da Cunha		MD	Hospital Metropolitano Odilon Behrens	Belo Horizonte, Minas Gerais, Brazil	Data collection	
Rafael Luis	Mol Guimaraes		MD	Hospital Metropolitano Odilon Behrens	Belo Horizonte, Minas Gerais, Brazil	Data collection	
Larissa	Moni Palma		MD	Hospital Metropolitano Odilon Behrens	Belo Horizonte, Minas Gerais, Brazil	Data collection	
Gustavo	Monteiro Vasconcelos		MD	Hospital Metropolitano Odilon Behrens	Belo Horizonte, Minas Gerais, Brazil	Data collection	
Vanessa	Moraes Marquez		MD	Hospital Metropolitano Odilon Behrens	Belo Horizonte, Minas Gerais, Brazil	Data collection	
Fernanda	Moreira Ballaris		MD	Hospital Metropolitano Odilon Behrens	Belo Horizonte, Minas Gerais, Brazil	Data collection	
Leticia	Moreira de Castro		MD	Hospital Metropolitano Odilon Behrens	Belo Horizonte, Minas Gerais, Brazil	Data collection	
Alice	Moreira dos Santos Marques		MD	Hospital Metropolitano Odilon Behrens	Belo Horizonte, Minas Gerais, Brazil	Data collection	
Thais	Moreira Martins da Costa		MD	Hospital Metropolitano Odilon Behrens	Belo Horizonte, Minas Gerais, Brazil	Data collection	

Supplemental Online Content: Nonauthor Collaborators
*First name, last name, and suffix (if applicable) are required and will appear in PubMed.

***First Name and Middle Initial(s)**	***Last Name**	***Suffix (eg, Jr, III)**	Academic Degrees	Institution	Location (city, state/province, country)	Role or Contribution, eg, chair, principal investigator	Group (if more than 1 Group listed in the byline) and/or Subgroup (eg, Steering Committee)
Clarisse	Moreira Ribeiro de Oliveira		MD	Hospital Metropolitano Odilon Behrens	Belo Horizonte, Minas Gerais, Brazil	Data collection	
Ana Carolina	Mota		MD	Hospital Metropolitano Odilon Behrens	Belo Horizonte, Minas Gerais, Brazil	Data collection	
Thales	Moura Camargo		MD	Hospital Metropolitano Odilon Behrens	Belo Horizonte, Minas Gerais, Brazil	Data collection	
Mateus Jorge	Nardelli		MD	Hospital Metropolitano Odilon Behrens	Belo Horizonte, Minas Gerais, Brazil	Data collection	
Bruno	Nunes		Medical Student	Hospital Metropolitano Odilon Behrens	Belo Horizonte, Minas Gerais, Brazil	Data collection	
Carolina	Oliveira Santos		MD	Hospital Metropolitano Odilon Behrens	Belo Horizonte, Minas Gerais, Brazil	Data collection	
Flavio Augusto	Paes de Oliveira		MD	Hospital Metropolitano Odilon Behrens	Belo Horizonte, Minas Gerais, Brazil	Data collection	
Gilmara	Paiva Quintao Costa		MD	Hospital Metropolitano Odilon Behrens	Belo Horizonte, Minas Gerais, Brazil	Data collection	
Vivian	Paiva Ribeiro		MD	Hospital Metropolitano Odilon Behrens	Belo Horizonte, Minas Gerais, Brazil	Data collection	
Marina	Paranhos Jalles		MD	Hospital Metropolitano Odilon Behrens	Belo Horizonte, Minas Gerais, Brazil	Data collection	
Marcia	Paulliny Soares Bahia		MD	Hospital Metropolitano Odilon Behrens	Belo Horizonte, Minas Gerais, Brazil	Data collection	
Anna Maria	Pereira		MD	Hospital Metropolitano Odilon Behrens	Belo Horizonte, Minas Gerais, Brazil	Data collection	
Benedicto	Pereira Barbosa		MD	Hospital Metropolitano Odilon Behrens	Belo Horizonte, Minas Gerais, Brazil	Data collection	
Andressa	Pi Rocha Reis		MD	Hospital Metropolitano Odilon Behrens	Belo Horizonte, Minas Gerais, Brazil	Data collection	
Andrei	Pinheiro Moura		MD	Hospital Metropolitano Odilon Behrens	Belo Horizonte, Minas Gerais, Brazil	Data collection	

Supplemental Online Content: Nonauthor Collaborators
*First name, last name, and suffix (if applicable) are required and will appear in PubMed.

*First Name and Middle Initial(s)	*Last Name	*Suffix (eg, Jr, III)	Academic Degrees	Institution	Location (city, state/province, country)	Role or Contribution, eg, chair, principal investigator	Group (if more than 1 Group listed in the byline) and/or Subgroup (eg, Steering Committee)
Gabriel	Prado Leao Teixeira		MD	Hospital Metropolitano Odilon Behrens	Belo Horizonte, Minas Gerais, Brazil	Data collection	
Laura	Rabelo Silva		MD	Hospital Metropolitano Odilon Behrens	Belo Horizonte, Minas Gerais, Brazil	Data collection	
Matheus	Rainato Zhouri		MD	Hospital Metropolitano Odilon Behrens	Belo Horizonte, Minas Gerais, Brazil	Data collection	
Isadora	Rangel		MD	Hospital Metropolitano Odilon Behrens	Belo Horizonte, Minas Gerais, Brazil	Data collection	
Isabella	Reis Santiago		MD	Hospital Metropolitano Odilon Behrens	Belo Horizonte, Minas Gerais, Brazil	Data collection	
Fillipe	Reis Silva		MD	Hospital Metropolitano Odilon Behrens	Belo Horizonte, Minas Gerais, Brazil	Data collection	
Giovanna	Ribas Passagli		MD	Hospital Metropolitano Odilon Behrens	Belo Horizonte, Minas Gerais, Brazil	Data collection	
Nadya	Ribeiro Correa		MD	Hospital Metropolitano Odilon Behrens	Belo Horizonte, Minas Gerais, Brazil	Data collection	
Pedro	Ribeiro de Jesus Almeida		MD	Hospital Metropolitano Odilon Behrens	Belo Horizonte, Minas Gerais, Brazil	Data collection	
Caio	Ribeiro Melki		MD	Hospital Metropolitano Odilon Behrens	Belo Horizonte, Minas Gerais, Brazil	Data collection	
Leonardo	Ribeiro Vieira		MD	Hospital Metropolitano Odilon Behrens	Belo Horizonte, Minas Gerais, Brazil	Data collection	
Felicio	Roberto Costa		MD	Hospital Metropolitano Odilon Behrens	Belo Horizonte, Minas Gerais, Brazil	Data collection	
Barbara	Rodrigues Toneli		MD	Hospital Metropolitano Odilon Behrens	Belo Horizonte, Minas Gerais, Brazil	Data collection	
Samuel	Rosa Ferreira		MD	Hospital Metropolitano Odilon Behrens	Belo Horizonte, Minas Gerais, Brazil	Data collection	
Yasmin	Rosa Storck Rocha Rodrigues		MD	Hospital Metropolitano Odilon Behrens	Belo Horizonte, Minas Gerais, Brazil	Data collection	

Supplemental Online Content: Nonauthor Collaborators
*First name, last name, and suffix (if applicable) are required and will appear in PubMed.

*First Name and Middle Initial(s)	*Last Name	*Suffix (eg, Jr, III)	Academic Degrees	Institution	Location (city, state/province, country)	Role or Contribution, eg, chair, principal investigator	Group (if more than 1 Group listed in the byline) and/or Subgroup (eg, Steering Committee)
Thiago	Ruiz Rodrigues Prestes		MD	Hospital Metropolitano Odilon Behrens	Belo Horizonte, Minas Gerais, Brazil	Data collection	
Leticia	Sampaio Barbosa Oliveira		MD	Hospital Metropolitano Odilon Behrens	Belo Horizonte, Minas Gerais, Brazil	Data collection	
Joao Pedro	Santana Vieira		MD	Hospital Metropolitano Odilon Behrens	Belo Horizonte, Minas Gerais, Brazil	Data collection	
Joao Pedro	Santana Vieira		MD	Hospital Metropolitano Odilon Behrens	Belo Horizonte, Minas Gerais, Brazil	Data collection	
Keytty Anny	Santos Oliveira		MD	Hospital Metropolitano Odilon Behrens	Belo Horizonte, Minas Gerais, Brazil	Data collection	
Gabriel	Santos Pessoa Isidoro		MD	Hospital Metropolitano Odilon Behrens	Belo Horizonte, Minas Gerais, Brazil	Data collection	
Rayan	Saude Barreto Ferreira		MD	Hospital Metropolitano Odilon Behrens	Belo Horizonte, Minas Gerais, Brazil	Data collection	
Luide	Scalioni Borges Dias		MD	Hospital Metropolitano Odilon Behrens	Belo Horizonte, Minas Gerais, Brazil	Data collection	
Gabriel	Seixas Carvalho		MD	Hospital Metropolitano Odilon Behrens	Belo Horizonte, Minas Gerais, Brazil	Data collection	
Ester	Sena Gomes de Almeida		MD	Hospital Metropolitano Odilon Behrens	Belo Horizonte, Minas Gerais, Brazil	Data collection	
Marina Luisa	Silva de Freitas		MD	Hospital Metropolitano Odilon Behrens	Belo Horizonte, Minas Gerais, Brazil	Data collection	
Cecilia	Silva de Paula Faria		MD	Hospital Metropolitano Odilon Behrens	Belo Horizonte, Minas Gerais, Brazil	Data collection	
Nathaly	Silva Silveira		MD	Hospital Metropolitano Odilon Behrens	Belo Horizonte, Minas Gerais, Brazil	Data collection	
Natalia	Silveira Cruz		MD	Hospital Metropolitano Odilon Behrens	Belo Horizonte, Minas Gerais, Brazil	Data collection	
Melina	Siqueira Machado		MD	Hospital Metropolitano Odilon Behrens	Belo Horizonte, Minas Gerais, Brazil	Data collection	

Supplemental Online Content: Nonauthor Collaborators
*First name, last name, and suffix (if applicable) are required and will appear in PubMed.

*First Name and Middle Initial(s)	*Last Name	*Suffix (eg, Jr, III)	Academic Degrees	Institution	Location (city, state/province, country)	Role or Contribution, eg, chair, principal investigator	Group (if more than 1 Group listed in the byline) and/or Subgroup (eg, Steering Committee)
Isabela	Soares Maia		MD	Hospital Metropolitano Odilon Behrens	Belo Horizonte, Minas Gerais, Brazil	Data collection	
Joao Pedro	Sousa Drumond		MD	Hospital Metropolitano Odilon Behrens	Belo Horizonte, Minas Gerais, Brazil	Data collection	
Diego	Sousa Fernandes		MD	Hospital Metropolitano Odilon Behrens	Belo Horizonte, Minas Gerais, Brazil	Data collection	
Flavia	Souza		MD	Hospital Metropolitano Odilon Behrens	Belo Horizonte, Minas Gerais, Brazil	Data collection	
Sofia	Souza Matoso		MD	Hospital Metropolitano Odilon Behrens	Belo Horizonte, Minas Gerais, Brazil	Data collection	
Frederico	Starling Leao		MD	Hospital Metropolitano Odilon Behrens	Belo Horizonte, Minas Gerais, Brazil	Data collection	
Vinicius	Torres Leite		MD	Hospital Metropolitano Odilon Behrens	Belo Horizonte, Minas Gerais, Brazil	Data collection	
Leticia	Ulhoa		MD	Hospital Metropolitano Odilon Behrens	Belo Horizonte, Minas Gerais, Brazil	Data collection	
Carolina	Vargas Duarte		MD	Hospital Metropolitano Odilon Behrens	Belo Horizonte, Minas Gerais, Brazil	Data collection	
Lucas	Vieira Chagas		MD	Hospital Metropolitano Odilon Behrens	Belo Horizonte, Minas Gerais, Brazil	Data collection	
Izabella	Vitor Lopes		MD	Hospital Metropolitano Odilon Behrens	Belo Horizonte, Minas Gerais, Brazil	Data collection	
Poliana	Xavier		MD	Hospital Metropolitano Odilon Behrens	Belo Horizonte, Minas Gerais, Brazil	Data collection	
Bruna	Xisto Mesquita de Oliveira		MD	Hospital Metropolitano Odilon Behrens	Belo Horizonte, Minas Gerais, Brazil	Data collection	
Aglair	Ferreira Muller		MD	Hospital Nossa Senhora da Conceicao	Porto Alegre, Rio Grande do Sul, Brazil	Data collection	
Diane	Alicia Tormes		MD	Hospital Nossa Senhora da Conceicao	Porto Alegre, Rio Grande do Sul, Brazil	Data collection	

Supplemental Online Content: Nonauthor Collaborators
*First name, last name, and suffix (if applicable) are required and will appear in PubMed.

*First Name and Middle Initial(s)	*Last Name	*Suffix (eg, Jr, III)	Academic Degrees	Institution	Location (city, state/province, country)	Role or Contribution, eg, chair, principal investigator	Group (if more than 1 Group listed in the byline) and/or Subgroup (eg, Steering Committee)
Ubirajara	Amaral Vinholes Filho		MD	Hospital Nossa Senhora da Conceicao	Porto Alegre, Rio Grande do Sul, Brazil	Data collection	
Yasmine	Badwan Mustafa		MD	Hospital Nossa Senhora da Conceicao	Porto Alegre, Rio Grande do Sul, Brazil	Data collection	
Cassia	Braga Grahl		MD	Hospital Nossa Senhora da Conceicao	Porto Alegre, Rio Grande do Sul, Brazil	Data collection	
Humberto Antonio	Bronzatto		MD	Hospital Nossa Senhora da Conceicao	Porto Alegre, Rio Grande do Sul, Brazil	Data collection	
Antonio	Claro dos Santos	Neto	MD	Hospital Nossa Senhora da Conceicao	Porto Alegre, Rio Grande do Sul, Brazil	Data collection	
Fernando	Da Silveira		MD	Hospital Nossa Senhora da Conceicao	Porto Alegre, Rio Grande do Sul, Brazil	Data collection	
Maria da Gloria	Delgado Ramos		MD	Hospital Nossa Senhora da Conceicao	Porto Alegre, Rio Grande do Sul, Brazil	Data collection	
Ana Cristina	Eickhoff		MD	Hospital Nossa Senhora da Conceicao	Porto Alegre, Rio Grande do Sul, Brazil	Data collection	
Alexandre	Freitas da Rosa		MD	Hospital Nossa Senhora da Conceicao	Porto Alegre, Rio Grande do Sul, Brazil	Data collection	
Giovana	Garziera		MD	Hospital Nossa Senhora da Conceicao	Porto Alegre, Rio Grande do Sul, Brazil	Data collection	
Juliana	Giacomelli Cao		MD	Hospital Nossa Senhora da Conceicao	Porto Alegre, Rio Grande do Sul, Brazil	Data collection	
Thamires	Helfer		MD	Hospital Nossa Senhora da Conceicao	Porto Alegre, Rio Grande do Sul, Brazil	Data collection	
Gustavo	Korssak Goncalves		MD	Hospital Nossa Senhora da Conceicao	Porto Alegre, Rio Grande do Sul, Brazil	Data collection	
Rafaela	Krewer		MD	Hospital Nossa Senhora da Conceicao	Porto Alegre, Rio Grande do Sul, Brazil	Data collection	
Luis Paulo	Kruse		MD	Hospital Nossa Senhora da Conceicao	Porto Alegre, Rio Grande do Sul, Brazil	Data collection	

Supplemental Online Content: Nonauthor Collaborators
*First name, last name, and suffix (if applicable) are required and will appear in PubMed.

*First Name and Middle Initial(s)	*Last Name	*Suffix (eg, Jr, III)	Academic Degrees	Institution	Location (city, state/province, country)	Role or Contribution, eg, chair, principal investigator	Group (if more than 1 Group listed in the byline) and/or Subgroup (eg, Steering Committee)
Rutielin	Machado Leite		MD	Hospital Nossa Senhora da Conceicao	Porto Alegre, Rio Grande do Sul, Brazil	Data collection	
Gabriel	Miranda		MD	Hospital Nossa Senhora da Conceicao	Porto Alegre, Rio Grande do Sul, Brazil	Data collection	
Tais	Pereira		Respiratory therapist	Hospital Nossa Senhora da Conceicao	Porto Alegre, Rio Grande do Sul, Brazil	Data collection	
Victoria	Porcher Simioni		MD	Hospital Nossa Senhora da Conceicao	Porto Alegre, Rio Grande do Sul, Brazil	Data collection	
Thais	Raimondi Sudbrack		MD	Hospital Nossa Senhora da Conceicao	Porto Alegre, Rio Grande do Sul, Brazil	Data collection	
Silas Eduardo	Reznicek		MD	Hospital Nossa Senhora da Conceicao	Porto Alegre, Rio Grande do Sul, Brazil	Data collection	
Camila	Ribas Stefanello		MD	Hospital Nossa Senhora da Conceicao	Porto Alegre, Rio Grande do Sul, Brazil	Data collection	
Ana Paula	Ribeiro Francisco		MD	Hospital Nossa Senhora da Conceicao	Porto Alegre, Rio Grande do Sul, Brazil	Data collection	
Paola	Rissardi		MD	Hospital Nossa Senhora da Conceicao	Porto Alegre, Rio Grande do Sul, Brazil	Data collection	
Adriano	Tavares Conceicao		MD	Hospital Nossa Senhora da Conceicao	Porto Alegre, Rio Grande do Sul, Brazil	Data collection	
Giovana	Thomasi Jahnke		MD	Hospital Nossa Senhora da Conceicao	Porto Alegre, Rio Grande do Sul, Brazil	Data collection	
Felipe	Veronese		MD	Hospital Nossa Senhora da Conceicao	Porto Alegre, Rio Grande do Sul, Brazil	Data collection	
Bruno	Vicenzo Thomas Bresolin		MD	Hospital Nossa Senhora da Conceicao	Porto Alegre, Rio Grande do Sul, Brazil	Data collection	
Lisiane	Vilar Santos		MD	Hospital Nossa Senhora da Conceicao	Porto Alegre, Rio Grande do Sul, Brazil	Data collection	

Supplemental Online Content: Nonauthor Collaborators
*First name, last name, and suffix (if applicable) are required and will appear in PubMed.

*First Name and Middle Initial(s)	*Last Name	*Suffix (eg, Jr, III)	Academic Degrees	Institution	Location (city, state/province, country)	Role or Contribution, eg, chair, principal investigator	Group (if more than 1 Group listed in the byline) and/or Subgroup (eg, Steering Committee)
Larissa	Zimmermann		MD	Hospital Nossa Senhora da Conceicao	Porto Alegre, Rio Grande do Sul, Brazil	Data collection	
Gustavo	Alvares da Cunha		MD	Hospital Regional Alto Vale	Rio do Sul, Santa Catarina, Brazil	Data collection	
Felipe	Brandao de Andrade		MD	Hospital Regional Alto Vale	Rio do Sul, Santa Catarina, Brazil	Data collection	
Mariani	Cristofolini		MD	Hospital Regional Alto Vale	Rio do Sul, Santa Catarina, Brazil	Data collection	
Phillippi Vinicius	do Livramento Pamplona		MS	Hospital Regional Alto Vale	Rio do Sul, Santa Catarina, Brazil	Data collection	
Luiz Diego	Grube		MD	Hospital Regional Alto Vale	Rio do Sul, Santa Catarina, Brazil	Data collection	
Jean	Kniss		MS	Hospital Regional Alto Vale	Rio do Sul, Santa Catarina, Brazil	Data collection	
João Paulo	Moraes		MD	Hospital Regional Alto Vale	Rio do Sul, Santa Catarina, Brazil	Data collection	
Fernando	Nicolas Lassalle Oliveira		MD	Hospital Regional Alto Vale	Rio do Sul, Santa Catarina, Brazil	Data collection	
Guilherme	Volpato		MS	Hospital Regional Alto Vale	Rio do Sul, Santa Catarina, Brazil	Data collection	
Guilherme	Torres Abi Ramia Chimelli		MD	Hospital Regional Alto Vale	Rio do Sul, Santa Catarina, Brazil	Regional Case Manager	
Paloma	Cristiny Siman		MD	Hospital Santa Cruz	Santa Cruz do Sul, Rio Grande do Sul, Brazil	Data collection	
Priscila Kellen	de Rezende		MD	Hospital Santa Cruz	Santa Cruz do Sul, Rio Grande do Sul, Brazil	Data collection	
Eugenio	Donato de Carvalho		MD	Hospital Santa Cruz	Santa Cruz do Sul, Rio Grande do Sul, Brazil	Data collection	
Robson	Gonçalves		MD	Hospital Santa Cruz	Santa Cruz do Sul, Rio Grande do Sul, Brazil	Data collection	

Supplemental Online Content: Nonauthor Collaborators
*First name, last name, and suffix (if applicable) are required and will appear in PubMed.

***First Name and Middle Initial(s)**	***Last Name**	***Suffix (eg, Jr, III)**	Academic Degrees	Institution	Location (city, state/province, country)	Role or Contribution, eg, chair, principal investigator	Group (if more than 1 Group listed in the byline) and/or Subgroup (eg, Steering Committee)
Juliano	Juliano Rathke		MD	Hospital Santa Cruz	Santa Cruz do Sul, Rio Grande do Sul, Brazil	Data collection	
Karoline	Kuczynski		MD	Hospital Santa Cruz	Santa Cruz do Sul, Rio Grande do Sul, Brazil	Data collection	
Walter	Limberger		MD	Hospital Santa Cruz	Santa Cruz do Sul, Rio Grande do Sul, Brazil	Data collection	
Tamires	Macedo da Silva		MD	Hospital Santa Cruz	Santa Cruz do Sul, Rio Grande do Sul, Brazil	Data collection	
Guilherme	Queiroz Fontana		MD	Hospital Santa Cruz	Santa Cruz do Sul, Rio Grande do Sul, Brazil	Data collection	
Priscila Maria	Uez		MD	Hospital Santa Cruz	Santa Cruz do Sul, Rio Grande do Sul, Brazil	Data collection	
Savio	Henrique dos Santos Torres		MD	Hospital Santo Antonio	Sinop, Mato Grosso, Brazil	Data collection	
Jofman	Amorim Leite e Silva		MD	Hospital Santo Antonio	Sinop, Mato Grosso, Brazil	Data collection	
Roger	Benet		MS	Hospital Santo Antonio	Sinop, Mato Grosso, Brazil	Data collection	
Leticia	Biguetti Nascimento		MD	Hospital Santo Antonio	Sinop, Mato Grosso, Brazil	Data collection	
Andrieli	Bouvier		Respiratory therapist	Hospital Santo Antonio	Sinop, Mato Grosso, Brazil	Data collection	
Matheus	Canton Assis		MD	Hospital Santo Antonio	Sinop, Mato Grosso, Brazil	Data collection	
Rafael	Chitolina		MD	Hospital Santo Antonio	Sinop, Mato Grosso, Brazil	Data collection	
Carolina	Coutinho		MD	Hospital Santo Antonio	Sinop, Mato Grosso, Brazil	Data collection	
Thiago	de Abreu		MD	Hospital Santo Antonio	Sinop, Mato Grosso, Brazil	Data collection	

Supplemental Online Content: Nonauthor Collaborators
*First name, last name, and suffix (if applicable) are required and will appear in PubMed.

***First Name and Middle Initial(s)**	***Last Name**	***Suffix (eg, Jr, III)**	Academic Degrees	Institution	Location (city, state/province, country)	Role or Contribution, eg, chair, principal investigator	Group (if more than 1 Group listed in the byline) and/or Subgroup (eg, Steering Committee)
Tiago	de Oliveira Ferreira		MD	Hospital Santo Antonio	Sinop, Mato Grosso, Brazil	Data collection	
Jamille	Dutra		MD	Hospital Santo Antonio	Sinop, Mato Grosso, Brazil	Data collection	
Fernanda	Fatima Luz		MD	Hospital Santo Antonio	Sinop, Mato Grosso, Brazil	Data collection	
Ana Lidia	Ferreira Ramos		Respiratory therapist	Hospital Santo Antonio	Sinop, Mato Grosso, Brazil	Data collection	
Raul	Ferreira Szabo		MD	Hospital Santo Antonio	Sinop, Mato Grosso, Brazil	Data collection	
Lucca	Goncalo de Castro Lima		MD	Hospital Santo Antonio	Sinop, Mato Grosso, Brazil	Data collection	
Kellen Thayanne	Hangai		MD	Hospital Santo Antonio	Sinop, Mato Grosso, Brazil	Data collection	
Lona	Hertha Klein		MD	Hospital Santo Antonio	Sinop, Mato Grosso, Brazil	Data collection	
Leticia	Kojima		MS	Hospital Santo Antonio	Sinop, Mato Grosso, Brazil	Data collection	
Andre	Luis Biesek		MD	Hospital Santo Antonio	Sinop, Mato Grosso, Brazil	Data collection	
Maite	Luise Zanette		MS	Hospital Santo Antonio	Sinop, Mato Grosso, Brazil	Data collection	
Rodrigo	Martins Alves		MD	Hospital Santo Antonio	Sinop, Mato Grosso, Brazil	Data collection	
Bruna Luiza	Oliveira Lima		MD	Hospital Santo Antonio	Sinop, Mato Grosso, Brazil	Data collection	
Joao Vicente	Rezende de Oliveira		MD	Hospital Santo Antonio	Sinop, Mato Grosso, Brazil	Data collection	
Paulo	Roberto de Miranda		MD	Hospital Santo Antonio	Sinop, Mato Grosso, Brazil	Data collection	

Supplemental Online Content: Nonauthor Collaborators
*First name, last name, and suffix (if applicable) are required and will appear in PubMed.

***First Name and Middle Initial(s)**	***Last Name**	***Suffix (eg, Jr, III)**	Academic Degrees	Institution	Location (city, state/province, country)	Role or Contribution, eg, chair, principal investigator	Group (if more than 1 Group listed in the byline) and/or Subgroup (eg, Steering Committee)
Laryssa	Semiguem		MD	Hospital Santo Antonio	Sinop, Mato Grosso, Brazil	Data collection	
Andressa	Tailine Jesus Lopes		Nurse	Hospital Santo Antonio	Sinop, Mato Grosso, Brazil	Data collection	
Marina Yuri	Takahara		MS	Hospital Santo Antonio	Sinop, Mato Grosso, Brazil	Data collection	
Mauricio	Bittencourt Macedo Rocha		MD	Hospital Sao Jose	Sao Jose, Santa Catarina, Brazil	Data collection	
Thor	Ferreira da Cruz da Cruz		MS	Hospital Sao Jose	Sao Jose, Santa Catarina, Brazil	Data collection	
Lucas	Koerich Severino		MD	Hospital Sao Jose	Sao Jose, Santa Catarina, Brazil	Data collection	
Isadorah	Nicaretta da Silva		MS	Hospital Sao Jose	Sao Jose, Santa Catarina, Brazil	Data collection	
Luisa	Sousa Eleutério de Azevedo		MD	Hospital Sao Jose	Sao Jose, Santa Catarina, Brazil	Data collection	
Luize	Stormovski de Araújo		MD	Hospital Sao Jose	Sao Jose, Santa Catarina, Brazil	Data collection	
Carlos Henrique	Aguiar Alves		MS	Hospital Sao Jose	Sao Jose, Santa Catarina, Brazil	Data collection	
Ester	Alegria Medina Franco Koch		MD	Hospital Sao Jose	Sao Jose, Santa Catarina, Brazil	Data collection	
Rodolfo	Antenow		MD	Hospital Sao Jose	Sao Jose, Santa Catarina, Brazil	Data collection	
Aline	Batista		MD	Hospital Sao Jose	Sao Jose, Santa Catarina, Brazil	Data collection	
Arnilda Clara	Beppler		MD	Hospital Sao Jose	Sao Jose, Santa Catarina, Brazil	Data collection	
Rafaela	Bernardi Ogliari		MS	Hospital Sao Jose	Sao Jose, Santa Catarina, Brazil	Data collection	

Supplemental Online Content: Nonauthor Collaborators
*First name, last name, and suffix (if applicable) are required and will appear in PubMed.

*First Name and Middle Initial(s)	*Last Name	*Suffix (eg, Jr, III)	Academic Degrees	Institution	Location (city, state/province, country)	Role or Contribution, eg, chair, principal investigator	Group (if more than 1 Group listed in the byline) and/or Subgroup (eg, Steering Committee)
Henrique	Bertotto		MD	Hospital Sao Jose	Sao Jose, Santa Catarina, Brazil	Data collection	
Jean Carlo	Bolsoni Rodrigues		MD	Hospital Sao Jose	Sao Jose, Santa Catarina, Brazil	Data collection	
Eduardo de Jesus	Bravo		MD	Hospital Sao Jose	Sao Jose, Santa Catarina, Brazil	Data collection	
Pedro	Búrigo Costa		MD	Hospital Sao Jose	Sao Jose, Santa Catarina, Brazil	Data collection	
Kaue	Caetano Bonacio		MD	Hospital Sao Jose	Sao Jose, Santa Catarina, Brazil	Data collection	
Eduardo Rosa	da Silva		MD	Hospital Sao Jose	Sao Jose, Santa Catarina, Brazil	Data collection	
Pedro	da Silva Rabelo		MD	Hospital Sao Jose	Sao Jose, Santa Catarina, Brazil	Data collection	
Giancarlo	De Antoni		MD	Hospital Sao Jose	Sao Jose, Santa Catarina, Brazil	Data collection	
Julia	de Carvalho Tiezzi		MD	Hospital Sao Jose	Sao Jose, Santa Catarina, Brazil	Data collection	
Plinio Henrique	de Castro Cezarino		MD	Hospital Sao Jose	Sao Jose, Santa Catarina, Brazil	Data collection	
Lourenco	de Miranda		MS	Hospital Sao Jose	Sao Jose, Santa Catarina, Brazil	Data collection	
Adriano Luiz	de Oliveira		MS	Hospital Sao Jose	Sao Jose, Santa Catarina, Brazil	Data collection	
Luiz Henrique	de Oliveira		MD	Hospital Sao Jose	Sao Jose, Santa Catarina, Brazil	Data collection	
Esthefânia	de Souza Maciel		MD	Hospital Sao Jose	Sao Jose, Santa Catarina, Brazil	Data collection	
Giulia	Dematé Tessarollo		MS	Hospital Sao Jose	Sao Jose, Santa Catarina, Brazil	Data collection	

Supplemental Online Content: Nonauthor Collaborators
*First name, last name, and suffix (if applicable) are required and will appear in PubMed.

*First Name and Middle Initial(s)	*Last Name	*Suffix (eg, Jr, III)	Academic Degrees	Institution	Location (city, state/province, country)	Role or Contribution, eg, chair, principal investigator	Group (if more than 1 Group listed in the byline) and/or Subgroup (eg, Steering Committee)
Rodrygo	Fanfa		MD	Hospital Sao Jose	Sao Jose, Santa Catarina, Brazil	Data collection	
João Camilo	Fernandes		MS	Hospital Sao Jose	Sao Jose, Santa Catarina, Brazil	Data collection	
Gabriel Henrique	Filgueira Ferrucci		MS	Hospital Sao Jose	Sao Jose, Santa Catarina, Brazil	Data collection	
Ana Clara	Flor da Costa		MD	Hospital Sao Jose	Sao Jose, Santa Catarina, Brazil	Data collection	
Victor	Francia Veloso Borges		MD	Hospital Sao Jose	Sao Jose, Santa Catarina, Brazil	Data collection	
Júlio César	Furlan		MS	Hospital Sao Jose	Sao Jose, Santa Catarina, Brazil	Data collection	
Flávia Karoline	Gamla Farias		MD	Hospital Sao Jose	Sao Jose, Santa Catarina, Brazil	Data collection	
Laura	Gazola Ugioni		MD	Hospital Sao Jose	Sao Jose, Santa Catarina, Brazil	Data collection	
Gabriel	Giassi Kochann		MD	Hospital Sao Jose	Sao Jose, Santa Catarina, Brazil	Data collection	
André Luiz	Hackbarth		MS	Hospital Sao Jose	Sao Jose, Santa Catarina, Brazil	Data collection	
Maria Luiza	Hensel		MD	Hospital Sao Jose	Sao Jose, Santa Catarina, Brazil	Data collection	
Roberta	Klein Serafini		MD	Hospital Sao Jose	Sao Jose, Santa Catarina, Brazil	Data collection	
Gabriel	Kochann		MD	Hospital Sao Jose	Sao Jose, Santa Catarina, Brazil	Data collection	
Diogo	Lima Costa		MD	Hospital Sao Jose	Sao Jose, Santa Catarina, Brazil	Regional Case Manager	
Bruna	Macena Cutti		MD	Hospital Sao Jose	Sao Jose, Santa Catarina, Brazil	Data collection	

Supplemental Online Content: Nonauthor Collaborators

*First name, last name, and suffix (if applicable) are required and will appear in PubMed.

*First Name and Middle Initial(s)	*Last Name	*Suffix (eg, Jr, III)	Academic Degrees	Institution	Location (city, state/province, country)	Role or Contribution, eg, chair, principal investigator	Group (if more than 1 Group listed in the byline) and/or Subgroup (eg, Steering Committee)
Laura	Machado Dalcin		MD	Hospital Sao Jose	Sao Jose, Santa Catarina, Brazil	Data collection	
Vitor Paulo	Marchioretto		MD	Hospital Sao Jose	Sao Jose, Santa Catarina, Brazil	Data collection	
Caroline	Marques		MD	Hospital Sao Jose	Sao Jose, Santa Catarina, Brazil	Data collection	
Geovane	Marques Andrade		MD	Hospital Sao Jose	Sao Jose, Santa Catarina, Brazil	Data collection	
Pedro Antônio	Meneghetti Spcart		MS	Hospital Sao Jose	Sao Jose, Santa Catarina, Brazil	Data collection	
João Victor	Meneses de Aguiar		MD	Hospital Sao Jose	Sao Jose, Santa Catarina, Brazil	Data collection	
Barbara Regina	Monteiro		MD	Hospital Sao Jose	Sao Jose, Santa Catarina, Brazil	Data collection	
Vinicius	Moreira		MS	Hospital Sao Jose	Sao Jose, Santa Catarina, Brazil	Data collection	
Bruno	Nahorny Ferreira		MD	Hospital Sao Jose	Sao Jose, Santa Catarina, Brazil	Data collection	
Lucas	Natale Cardoso		MD	Hospital Sao Jose	Sao Jose, Santa Catarina, Brazil	Data collection	
Gustavo	Nogueira Schincariol Vicente		MD	Hospital Sao Jose	Sao Jose, Santa Catarina, Brazil	Data collection	
Ana Clara	Pilatti		MD	Hospital Sao Jose	Sao Jose, Santa Catarina, Brazil	Data collection	
Aline	Ribeiro Caminha		MD	Hospital Sao Jose	Sao Jose, Santa Catarina, Brazil	Data collection	
Renata	Rossi		MD	Hospital Sao Jose	Sao Jose, Santa Catarina, Brazil	Data collection	
Francisco	Schossler Loss		MD	Hospital Sao Jose	Sao Jose, Santa Catarina, Brazil	Data collection	

Supplemental Online Content: Nonauthor Collaborators
*First name, last name, and suffix (if applicable) are required and will appear in PubMed.

***First Name and Middle Initial(s)**	***Last Name**	***Suffix (eg, Jr, III)**	Academic Degrees	Institution	Location (city, state/province, country)	Role or Contribution, eg, chair, principal investigator	Group (if more than 1 Group listed in the byline) and/or Subgroup (eg, Steering Committee)
Laura	Silva Padiha		MS	Hospital Sao Jose	Sao Jose, Santa Catarina, Brazil	Data collection	
Daniele Carolina	Silveira Battaglini		MD	Hospital Sao Jose	Sao Jose, Santa Catarina, Brazil	Data collection	
Camila	Simas		Nurse	Hospital Sao Jose	Sao Jose, Santa Catarina, Brazil	Data collection	
Luiz Gustavo	Souza Cardozo		MD	Hospital Sao Jose	Sao Jose, Santa Catarina, Brazil	Data collection	
Marcos Vinicius	Souza Rafaeli		MD	Hospital Sao Jose	Sao Jose, Santa Catarina, Brazil	Data collection	
Rubia Ilidia	Tambones Galdino		MD	Hospital Sao Jose	Sao Jose, Santa Catarina, Brazil	Data collection	
Luiz Augusto	Tenconi		MD	Hospital Sao Jose	Sao Jose, Santa Catarina, Brazil	Data collection	
José Vítor	Tondo		MD	Hospital Sao Jose	Sao Jose, Santa Catarina, Brazil	Data collection	
Sarah	Van Den Berge		MD	Hospital Sao Jose	Sao Jose, Santa Catarina, Brazil	Data collection	
Gabriel Augusto	Vieira		MS	Hospital Sao Jose	Sao Jose, Santa Catarina, Brazil	Data collection	
Gabriel	Vieira		MS	Hospital Sao Jose	Sao Jose, Santa Catarina, Brazil	Data collection	
Ana Victória	Zambonetti Mendry		MD	Hospital Sao Jose	Sao Jose, Santa Catarina, Brazil	Data collection	
Nathalia	Zanotto Bernardi		MD	Hospital Sao Jose	Sao Jose, Santa Catarina, Brazil	Data collection	
Felipe Renato	Barrachini Steffen		MD	Hospital Sao Lucas da Pontificia Universidade Catolica do RS	Porto Alegre, Rio Grande do Sul, Brazil	Data collection	
Rozeli	Biedrzycki		MD	Hospital Sao Lucas da Pontificia Universidade Catolica do RS	Porto Alegre, Rio Grande do Sul, Brazil	Data collection	

Supplemental Online Content: Nonauthor Collaborators
*First name, last name, and suffix (if applicable) are required and will appear in PubMed.

***First Name and Middle Initial(s)**	***Last Name**	***Suffix (eg, Jr, III)**	Academic Degrees	Institution	Location (city, state/province, country)	Role or Contribution, eg, chair, principal investigator	Group (if more than 1 Group listed in the byline) and/or Subgroup (eg, Steering Committee)
Francine	Camazzola Modena		MD	Hospital Sao Lucas da Pontificia Universidade Catolica do RS	Porto Alegre, Rio Grande do Sul, Brazil	Data collection	
Gabriel	Dias de Souza		MD	Hospital Sao Lucas da Pontificia Universidade Catolica do RS	Porto Alegre, Rio Grande do Sul, Brazil	Data collection	
Pietro	Durigan		MD	Hospital Sao Lucas da Pontificia Universidade Catolica do RS	Porto Alegre, Rio Grande do Sul, Brazil	Data collection	
Bárbara Horbach	Horbach		MD	Hospital Sao Lucas da Pontificia Universidade Catolica do RS	Porto Alegre, Rio Grande do Sul, Brazil	Data collection	
Caroline Locatelli Da Silva	Locatelli Da Silva		MD	Hospital Sao Lucas da Pontificia Universidade Catolica do RS	Porto Alegre, Rio Grande do Sul, Brazil	Data collection	
Marcelo	Menegotto Donadel		MD	Hospital Sao Lucas da Pontificia Universidade Catolica do RS	Porto Alegre, Rio Grande do Sul, Brazil	Data collection	
Guilherme Henrique	Muller		MD	Hospital Sao Lucas da Pontificia Universidade Catolica do RS	Porto Alegre, Rio Grande do Sul, Brazil	Data collection	
Guilherme	Paza Ferreira		MS	Hospital Sao Lucas da Pontificia Universidade Catolica do RS	Porto Alegre, Rio Grande do Sul, Brazil	Data collection	
Leticia	Petry Nicodem		MD	Hospital Sao Lucas da Pontificia Universidade Catolica do RS	Porto Alegre, Rio Grande do Sul, Brazil	Data collection	
João Pedro	Pizzato Sidou		MD	Hospital Sao Lucas da Pontificia Universidade Catolica do RS	Porto Alegre, Rio Grande do Sul, Brazil	Data collection	
Raquel	Potrich Zen		MD	Hospital Sao Lucas da Pontificia Universidade Catolica do RS	Porto Alegre, Rio Grande do Sul, Brazil	Data collection	
Erida Thayna	Ribeiro		MD	Hospital Sao Lucas da Pontificia Universidade Catolica do RS	Porto Alegre, Rio Grande do Sul, Brazil	Data collection	
Laura Diana	Ribeiro dos Anjos Muianga Alafo		MD	Hospital Sao Lucas da Pontificia Universidade Catolica do RS	Porto Alegre, Rio Grande do Sul, Brazil	Data collection	
Eduarda Rosa Fernandes	Rosa Fernandes		MD	Hospital Sao Lucas da Pontificia Universidade Catolica do RS	Porto Alegre, Rio Grande do Sul, Brazil	Data collection	
Victória	Sartor Poloni		MD	Hospital Sao Lucas da Pontificia Universidade Catolica do RS	Porto Alegre, Rio Grande do Sul, Brazil	Data collection	

Supplemental Online Content: Nonauthor Collaborators
*First name, last name, and suffix (if applicable) are required and will appear in PubMed.

*First Name and Middle Initial(s)	*Last Name	*Suffix (eg, Jr, III)	Academic Degrees	Institution	Location (city, state/province, country)	Role or Contribution, eg, chair, principal investigator	Group (if more than 1 Group listed in the byline) and/or Subgroup (eg, Steering Committee)
Luiz Felipe	Schmidt Birk		MD	Hospital Sao Lucas da Pontificia Universidade Catolica do RS	Porto Alegre, Rio Grande do Sul, Brazil	Data collection	
Lisiane	Vila Santos		MD	Hospital Sao Lucas da Pontificia Universidade Catolica do RS	Porto Alegre, Rio Grande do Sul, Brazil	Data collection	
Veronica	Westphal		MD	Hospital Sao Lucas da Pontificia Universidade Catolica do RS	Porto Alegre, Rio Grande do Sul, Brazil	Data collection	
Hian	Vechiato Betoni		Medical Student	Instituto central do HCFMUSP	Sao Paulo, Sao Paulo, Brazil	Data collection	
Giovanna	Cardoso de Oliveira		Medical Student	Instituto central do HCFMUSP	Sao Paulo, Sao Paulo, Brazil	Data collection	
Gabriel	Petrin Alonso Silva		Medical Student	Instituto central do HCFMUSP	Sao Paulo, Sao Paulo, Brazil	Data collection	
Mayara	Assis Kovachich de Oliveira		MD	Instituto central do HCFMUSP	Sao Paulo, Sao Paulo, Brazil	Data collection	
Bruna	Bandeira de Mello Oliveira		MD	Instituto central do HCFMUSP	Sao Paulo, Sao Paulo, Brazil	Data collection	
Laura	Brenha Ribeiro Martins		MD	Instituto central do HCFMUSP	Sao Paulo, Sao Paulo, Brazil	Data collection	
Mariana	Cury Sobreira		Respiratory therapist	Instituto central do HCFMUSP	Sao Paulo, Sao Paulo, Brazil	Data collection	
Fabio Augusto	da Rocha Specian		MD	Instituto central do HCFMUSP	Sao Paulo, Sao Paulo, Brazil	Data collection	
Camila	de Castello Branco Boccato		MD	Instituto central do HCFMUSP	Sao Paulo, Sao Paulo, Brazil	Data collection	
Mateus	de Castro dos Santos		MD	Instituto central do HCFMUSP	Sao Paulo, Sao Paulo, Brazil	Data collection	
Fabrício Henrique	de Oliveira Almeida		Physiotherapist	Instituto central do HCFMUSP	Sao Paulo, Sao Paulo, Brazil	Data collection	

Supplemental Online Content: Nonauthor Collaborators
*First name, last name, and suffix (if applicable) are required and will appear in PubMed.

*First Name and Middle Initial(s)	*Last Name	*Suffix (eg, Jr, III)	Academic Degrees	Institution	Location (city, state/province, country)	Role or Contribution, eg, chair, principal investigator	Group (if more than 1 Group listed in the byline) and/or Subgroup (eg, Steering Committee)
Felipe	de Souza Cabral		MD	Instituto central do HCFMUSP	Sao Paulo, Sao Paulo, Brazil	Data collection	
Tauani	dos Santos Cordeiro		MD	Instituto central do HCFMUSP	Sao Paulo, Sao Paulo, Brazil	Data collection	
Luiz Rodolfo	Egydio de Cerqueira Cesar		MD	Instituto central do HCFMUSP	Sao Paulo, Sao Paulo, Brazil	Data collection	
Michelle	Garcia Ferreira de Oliveira		MD	Instituto central do HCFMUSP	Sao Paulo, Sao Paulo, Brazil	Data collection	
Lucas	Leopoldino Resende de Oliveira		MD	Instituto central do HCFMUSP	Sao Paulo, Sao Paulo, Brazil	Data collection	
Raquel	Lins das Chagas Lima		MD	Instituto central do HCFMUSP	Sao Paulo, Sao Paulo, Brazil	Data collection	
Julia	Magalhaes Dorn de Carvalho		MD	Instituto central do HCFMUSP	Sao Paulo, Sao Paulo, Brazil	Data collection	
Helena	Ribeiro Aiello Amat		MD	Instituto central do HCFMUSP	Sao Paulo, Sao Paulo, Brazil	Data collection	
Mariana	Rodrigues Kisling Ávila		MD	Instituto central do HCFMUSP	Sao Paulo, Sao Paulo, Brazil	Data collection	
William	Simões Rangel Junior		MD	Instituto central do HCFMUSP	Sao Paulo, Sao Paulo, Brazil	Data collection	
Andre	Abou Haidar		MD	Instituto central do HCFMUSP	Sao Paulo, Sao Paulo, Brazil	Data collection	
Thaís	Alves Assumpção		MD	Instituto central do HCFMUSP	Sao Paulo, Sao Paulo, Brazil	Data collection	
Rosanita	Alves Rocha		MD	Instituto central do HCFMUSP	Sao Paulo, Sao Paulo, Brazil	Data collection	
Laura	Andrade		Physiotera pist	Instituto central do HCFMUSP	Sao Paulo, Sao Paulo, Brazil	Data collection	

Supplemental Online Content: Nonauthor Collaborators
*First name, last name, and suffix (if applicable) are required and will appear in PubMed.

*First Name and Middle Initial(s)	*Last Name	*Suffix (eg, Jr, III)	Academic Degrees	Institution	Location (city, state/province, country)	Role or Contribution, eg, chair, principal investigator	Group (if more than 1 Group listed in the byline) and/or Subgroup (eg, Steering Committee)
Pedro Henrique	Andrade Araújo Salvatore Barletta		MD	Instituto central do HCFMUSP	Sao Paulo, Sao Paulo, Brazil	Data collection	
Mateus	Andrade Bomfim Machado		MD	Instituto central do HCFMUSP	Sao Paulo, Sao Paulo, Brazil	Data collection	
Amarilis	Araújo Domingues		MS	Instituto central do HCFMUSP	Sao Paulo, Sao Paulo, Brazil	Data collection	
Joao Marcelo	Araujo Vieira		Physiotera pist	Instituto central do HCFMUSP	Sao Paulo, Sao Paulo, Brazil	Data collection	
Giovanna	Babikian Costa		MD	Instituto central do HCFMUSP	Sao Paulo, Sao Paulo, Brazil	Data collection	
Eduarda	Baccarin Ferrari		MD	Instituto central do HCFMUSP	Sao Paulo, Sao Paulo, Brazil	Data collection	
Janaína Aparecida	Baragão Moretoni		MD	Instituto central do HCFMUSP	Sao Paulo, Sao Paulo, Brazil	Data collection	
Andrea	Beatrice		MD	Instituto central do HCFMUSP	Sao Paulo, Sao Paulo, Brazil	Data collection	
Gustavo	Biz Martins		MD	Instituto central do HCFMUSP	Sao Paulo, Sao Paulo, Brazil	Data collection	
Marcos	Bozzolo		MD	Instituto central do HCFMUSP	Sao Paulo, Sao Paulo, Brazil	Data collection	
Mateus	Braga Birello		MD	Instituto central do HCFMUSP	Sao Paulo, Sao Paulo, Brazil	Data collection	
Henrique	Brito Silveira		MS	Instituto central do HCFMUSP	Sao Paulo, Sao Paulo, Brazil	Data collection	
Eduarda	Campos Menegaço		MD	Instituto central do HCFMUSP	Sao Paulo, Sao Paulo, Brazil	Data collection	
Nathalia	Campos Rodrigues		MD	Instituto central do HCFMUSP	Sao Paulo, Sao Paulo, Brazil	Data collection	

Supplemental Online Content: Nonauthor Collaborators
*First name, last name, and suffix (if applicable) are required and will appear in PubMed.

*First Name and Middle Initial(s)	*Last Name	*Suffix (eg, Jr, III)	Academic Degrees	Institution	Location (city, state/province, country)	Role or Contribution, eg, chair, principal investigator	Group (if more than 1 Group listed in the byline) and/or Subgroup (eg, Steering Committee)
Barbara	Canto Estevam		MD	Instituto central do HCFMUSP	Sao Paulo, Sao Paulo, Brazil	Data collection	
Giovana	Carnielli		Physiotera pist	Instituto central do HCFMUSP	Sao Paulo, Sao Paulo, Brazil	Data collection	
Guilherme	Carvalho		MD	Instituto central do HCFMUSP	Sao Paulo, Sao Paulo, Brazil	Data collection	
Alisson	Carvalho de Freitas		MD	Instituto central do HCFMUSP	Sao Paulo, Sao Paulo, Brazil	Data collection	
Amanda	Castilho Souza Balbino		MD	Instituto central do HCFMUSP	Sao Paulo, Sao Paulo, Brazil	Data collection	
João Carlos	Clarck Barros		MS	Instituto central do HCFMUSP	Sao Paulo, Sao Paulo, Brazil	Data collection	
Karine	Corcione Turke		MD	Instituto central do HCFMUSP	Sao Paulo, Sao Paulo, Brazil	Data collection	
José Henrique	Cordeiro e Silva		MD	Instituto central do HCFMUSP	Sao Paulo, Sao Paulo, Brazil	Data collection	
Isabela	Correa Areias		MD	Instituto central do HCFMUSP	Sao Paulo, Sao Paulo, Brazil	Data collection	
Tereza	Costa Gomes		MD	Instituto central do HCFMUSP	Sao Paulo, Sao Paulo, Brazil	Data collection	
Túlio	Couto Medeiros		MD	Instituto central do HCFMUSP	Sao Paulo, Sao Paulo, Brazil	Data collection	
Gilberto Carlos	de Almeida		MD	Instituto central do HCFMUSP	Sao Paulo, Sao Paulo, Brazil	Data collection	
Arthur	de Campos Soares		MD	Instituto central do HCFMUSP	Sao Paulo, Sao Paulo, Brazil	Data collection	
Jade Lara	de melo		Physiotera pist	Instituto central do HCFMUSP	Sao Paulo, Sao Paulo, Brazil	Data collection	
Vinicius	Fernandes Oliveira		MD	Instituto central do HCFMUSP	Sao Paulo, Sao Paulo, Brazil	Data collection	

Supplemental Online Content: Nonauthor Collaborators
*First name, last name, and suffix (if applicable) are required and will appear in PubMed.

*First Name and Middle Initial(s)	*Last Name	*Suffix (eg, Jr, III)	Academic Degrees	Institution	Location (city, state/province, country)	Role or Contribution, eg, chair, principal investigator	Group (if more than 1 Group listed in the byline) and/or Subgroup (eg, Steering Committee)
Noemia	Ferreira		MD	Instituto central do HCFMUSP	Sao Paulo, Sao Paulo, Brazil	Data collection	
Yasmine	Filippo		Physiotera pist	Instituto central do HCFMUSP	Sao Paulo, Sao Paulo, Brazil	Data collection	
João Gabriel	Fragoso Dias		MD	Instituto central do HCFMUSP	Sao Paulo, Sao Paulo, Brazil	Data collection	
Juliana	Galvão		MD	Instituto central do HCFMUSP	Sao Paulo, Sao Paulo, Brazil	Data collection	
Thiago Francisco	Gava		Nurse	Instituto central do HCFMUSP	Sao Paulo, Sao Paulo, Brazil	Data collection	
Fernanda	Greco		Physiotera pist	Instituto central do HCFMUSP	Sao Paulo, Sao Paulo, Brazil	Data collection	
Gabriel	Guimarães Mandaji		MD	Instituto central do HCFMUSP	Sao Paulo, Sao Paulo, Brazil	Data collection	
Heloisa	Heloisa Zogheib		MS	Instituto central do HCFMUSP	Sao Paulo, Sao Paulo, Brazil	Data collection	
Amanda	Kaori Ito		MD	Instituto central do HCFMUSP	Sao Paulo, Sao Paulo, Brazil	Data collection	
Man Hong	Lee		MD	Instituto central do HCFMUSP	Sao Paulo, Sao Paulo, Brazil	Data collection	
Luana	Luana Souza		Physiotera pist	Instituto central do HCFMUSP	Sao Paulo, Sao Paulo, Brazil	Data collection	
Gabriel	Maia de Albuquerque Costa		MD	Instituto central do HCFMUSP	Sao Paulo, Sao Paulo, Brazil	Data collection	
Rômulo Iago	Maniçoba Alves		MD	Instituto central do HCFMUSP	Sao Paulo, Sao Paulo, Brazil	Data collection	
Gabriel	Martinez		MD	Instituto central do HCFMUSP	Sao Paulo, Sao Paulo, Brazil	Data collection	
Pedro	Mazzilli Suplicy		MD	Instituto central do HCFMUSP	Sao Paulo, Sao Paulo, Brazil	Data collection	

Supplemental Online Content: Nonauthor Collaborators
*First name, last name, and suffix (if applicable) are required and will appear in PubMed.

***First Name and Middle Initial(s)**	***Last Name**	***Suffix (eg, Jr, III)**	Academic Degrees	Institution	Location (city, state/province, country)	Role or Contribution, eg, chair, principal investigator	Group (if more than 1 Group listed in the byline) and/or Subgroup (eg, Steering Committee)
Juliana	Meireles do Nascimento Macedo		MD	Instituto central do HCFMUSP	Sao Paulo, Sao Paulo, Brazil	Data collection	
Caroline	Morales		Physiotherapist	Instituto central do HCFMUSP	Sao Paulo, Sao Paulo, Brazil	Data collection	
Patrícia	Moura		Physiotherapist	Instituto central do HCFMUSP	Sao Paulo, Sao Paulo, Brazil	Data collection	
Victor	Navarro Jordão		MD	Instituto central do HCFMUSP	Sao Paulo, Sao Paulo, Brazil	Data collection	
Heraldo	Possolo		MD, Phd	Instituto central do HCFMUSP	Sao Paulo, Sao Paulo, Brazil	Investigator	
Carlos	Netto		MD	Instituto central do HCFMUSP	Sao Paulo, Sao Paulo, Brazil	Data collection	
Angélica Mirian	Nunes Toledo		MD	Instituto central do HCFMUSP	Sao Paulo, Sao Paulo, Brazil	Data collection	
Yan Pedro	Pagnard		MD	Instituto central do HCFMUSP	Sao Paulo, Sao Paulo, Brazil	Data collection	
Victor	Paro da Cunha		MD	Instituto central do HCFMUSP	Sao Paulo, Sao Paulo, Brazil	Data collection	
Caroline	Pelegrini Correia		MD	Instituto central do HCFMUSP	Sao Paulo, Sao Paulo, Brazil	Data collection	
Victor	Pessini		MD	Instituto central do HCFMUSP	Sao Paulo, Sao Paulo, Brazil	Data collection	
Giovanna	Placco Bezerra		Physiotherapist	Instituto central do HCFMUSP	Sao Paulo, Sao Paulo, Brazil	Data collection	
Célia	Raiany Ferreira de Farias		MD	Instituto central do HCFMUSP	Sao Paulo, Sao Paulo, Brazil	Data collection	
Armando Henrique	Ramos Minucci		MD	Instituto central do HCFMUSP	Sao Paulo, Sao Paulo, Brazil	Data collection	

Supplemental Online Content: Nonauthor Collaborators
*First name, last name, and suffix (if applicable) are required and will appear in PubMed.

***First Name and Middle Initial(s)**	***Last Name**	***Suffix (eg, Jr, III)**	Academic Degrees	Institution	Location (city, state/province, country)	Role or Contribution, eg, chair, principal investigator	Group (if more than 1 Group listed in the byline) and/or Subgroup (eg, Steering Committee)
Daniel	Ranzeiro de Bragança Aylmer		MD	Instituto central do HCFMUSP	Sao Paulo, Sao Paulo, Brazil	Data collection	
Eziquiel	Reginato Filho		MD	Instituto central do HCFMUSP	Sao Paulo, Sao Paulo, Brazil	Data collection	
João Pedro	Resende Scapim		MD	Instituto central do HCFMUSP	Sao Paulo, Sao Paulo, Brazil	Data collection	
Cesar	Ribeiro Filadelfo		MD	Instituto central do HCFMUSP	Sao Paulo, Sao Paulo, Brazil	Data collection	
Carlos	Roberto		MD	Instituto central do HCFMUSP	Sao Paulo, Sao Paulo, Brazil	Data collection	
Sabrina	Sabrina Ribeiro		MD	Instituto central do HCFMUSP	Sao Paulo, Sao Paulo, Brazil	Data collection	
Adara	Saito Goes		Physiotera pist	Instituto central do HCFMUSP	Sao Paulo, Sao Paulo, Brazil	Data collection	
Marcel	Santos		MD	Instituto central do HCFMUSP	Sao Paulo, Sao Paulo, Brazil	Data collection	
Rodrigo Matheus	Santos Alves		MD	Instituto central do HCFMUSP	Sao Paulo, Sao Paulo, Brazil	Data collection	
Natália	Simões Gambini		MD	Instituto central do HCFMUSP	Sao Paulo, Sao Paulo, Brazil	Data collection	
Beatriz	Soletti Pereira		MD	Instituto central do HCFMUSP	Sao Paulo, Sao Paulo, Brazil	Data collection	
Bruna	Souza Marques		MD	Instituto central do HCFMUSP	Sao Paulo, Sao Paulo, Brazil	Data collection	
Mateus	Teixeira Candido		MD	Instituto central do HCFMUSP	Sao Paulo, Sao Paulo, Brazil	Data collection	
Gustavo	Toledo de Freitas		MD	Instituto central do HCFMUSP	Sao Paulo, Sao Paulo, Brazil	Data collection	
Estela	Varella		MS	Instituto central do HCFMUSP	Sao Paulo, Sao Paulo, Brazil	Data collection	

Supplemental Online Content: Nonauthor Collaborators
*First name, last name, and suffix (if applicable) are required and will appear in PubMed.

*First Name and Middle Initial(s)	*Last Name	*Suffix (eg, Jr, III)	Academic Degrees	Institution	Location (city, state/province, country)	Role or Contribution, eg, chair, principal investigator	Group (if more than 1 Group listed in the byline) and/or Subgroup (eg, Steering Committee)
Thales	Vassoler Mendes da Silva		MD	Instituto central do HCFMUSP	Sao Paulo, Sao Paulo, Brazil	Data collection	
Igor	Veiga Silverio		MD	Instituto central do HCFMUSP	Sao Paulo, Sao Paulo, Brazil	Data collection	
Viktoria	Weihermann		MD	Instituto central do HCFMUSP	Sao Paulo, Sao Paulo, Brazil	Data collection	
Joniel José	Wiebbelling da Silva		MD	Instituto central do HCFMUSP	Sao Paulo, Sao Paulo, Brazil	Data collection	
Geovane	Wiebelling		MD	Instituto central do HCFMUSP	Sao Paulo, Sao Paulo, Brazil	Data collection	
Thiago	Ximenes Ferraz		MD	Instituto central do HCFMUSP	Sao Paulo, Sao Paulo, Brazil	Data collection	
Lucca	Zampolli		MS	Instituto central do HCFMUSP	Sao Paulo, Sao Paulo, Brazil	Data collection	
Cristian	Amaral Pereira		MD	Unidade de Pronto Atendimento - Lajeado	Lajeado, Rio Grande do Sul, Brazil	Data collection	
Henrique Augusto	Barcelos Knaack		MD	Unidade de Pronto Atendimento - Lajeado	Lajeado, Rio Grande do Sul, Brazil	Data collection	
Vitor	Cremonese Zanella		MD	Unidade de Pronto Atendimento - Lajeado	Lajeado, Rio Grande do Sul, Brazil	Data collection	
Fernanda	Giacomini		MD	Unidade de Pronto Atendimento - Lajeado	Lajeado, Rio Grande do Sul, Brazil	Data collection	
Eduardo	Junkherr Salgueiro		MD	Unidade de Pronto Atendimento - Lajeado	Lajeado, Rio Grande do Sul, Brazil	Data collection	
Augusto	Lengler Konrath		MD	Unidade de Pronto Atendimento - Lajeado	Lajeado, Rio Grande do Sul, Brazil	Data collection	
Marciele	Pazinatto		MD	Unidade de Pronto Atendimento - Lajeado	Lajeado, Rio Grande do Sul, Brazil	Data collection	
Vanessa	Reis		MD	Unidade de Pronto Atendimento - Lajeado	Lajeado, Rio Grande do Sul, Brazil	Data collection	
