# Supplementary material for: Ketamine, Etomidate, and Mortality in Emergency Department Intubations
Source: JAMA Netw Open. 2025 Dec 15;8(12):e2548060. doi: 10.1001/jamanetworkopen.2025.48060 (PMC12706683; doi:10.1001/jamanetworkopen.2025.48060)
Supplement: Supplement 3. — Data Sharing Statement [file jamanetwopen-e2548060-s003.pdf]

Data Sharing Statement

Maia. Ketamine, Etomidate, and Mortality in Emergency Department Intubations. *JAMA Netw Open*. Published December 15, 2025. doi:10.1001/jamanetworkopen.2025.48060

Data
Data available: Yes
Data types: Deidentified participant data, Data dictionary
How to access data: Data was abstracted from the published articles and is available upon request. Derived data, the study protocol, statistical analysis plan, and analytic code can also be provided on request, promoting transparency and reproducibility.
When available: With publication

Supporting Documents
Document types: None

Additional Information
Who can access the data: Data was abstracted from the published articles and is available upon request. Derived data, the study protocol, statistical analysis plan, and analytic code can also be provided on request, promoting transparency and reproducibility.
Types of analyses: Data was abstracted from the published articles and is available upon request. Derived data, the study protocol, statistical analysis plan, and analytic code can also be provided on request, promoting transparency and reproducibility.
Mechanisms of data availability: Data was abstracted from the published articles and is available upon request. Derived data, the study protocol, statistical analysis plan, and analytic code can also be provided on request, promoting transparency and reproducibility.
